# Flow diversion of ruptured intracranial aneurysms: a single-center study with a standardized antithrombotic treatment protocol

**DOI:** 10.1007/s00701-024-06029-7

**Published:** 2024-03-11

**Authors:** Anni Rantamo, Camille Gallé, Jussi Numminen, Jyri Virta, Päivi Tanskanen, Ann-Christine Lindroos, Julio Resendiz-Nieves, Martin Lehecka, Mika Niemelä, Roel Haeren, Rahul Raj

**Affiliations:** 1 Department of Neurosurgery, Helsinki University Hospital and University of Helsinki, Haartmaninkatu 4, Po Box 320, 00029 HUS Helsinki, Finland; 2 Department of Neurosurgery, Maastricht University Medical Center, PO box 5800, 6202 AZ Maastricht, The Netherlands; 3 Anaesthesiology and Intensive Care Medicine, Helsinki University Hospital, University of Helsinki, Helsinki, Finland

**Keywords:** Flow diversion, Intracranial aneurysm, Flow diverter, Subarachnoid hemorrhage, Intensive care, Antithrombotic medication, Dual antiplatelet therapy

## Abstract

**Background:**

The use of antithrombotic medication following acute flow diversion for a ruptured intracranial aneurysm (IA) is challenging with no current guidelines. We investigated the incidence of treatment-related complications and patient outcomes after flow diversion for a ruptured IA before and after the implementation of a standardized antithrombotic medication protocol.

**Methods:**

We conducted a single-center retrospective study including consecutive patients treated for acutely ruptured IAs with flow diversion during 2015–2023. We divided the patients into two groups: those treated before the implementation of the protocol (pre-protocol) and those treated after the implementation of the protocol (post-protocol). The primary outcomes were hemorrhagic and ischemic complications. A secondary outcome was clinical outcome using the modified Ranking Scale (mRS).

**Results:**

Totally 39 patients with 40 ruptured IAs were treated with flow diversion (69% pre-protocol, 31% post-protocol). The patient mean age was 55 years, 62% were female, 63% of aneurysms were in the posterior circulation, 92% of aneurysms were non-saccular, and 44% were in poor grade on admission. Treatment differences included the use of glycoprotein IIb/IIIa inhibitors (pre-group 48% vs. post-group 100%), and the use of early dual antiplatelets (pre-group 44% vs. 92% post-group). The incidence of ischemic complications was 37% and 42% and the incidence of hemorrhagic complications was 30% and 33% in the pre- and post-groups, respectively, with no between-group differences. There were three (11%) aneurysm re-ruptures in the pre-group and none in the post-group. There were no differences in mortality or mRS 0–2 between the groups at 6 months.

**Conclusion:**

We found no major differences in the incidence of ischemic or hemorrhagic complications after the implementation of a standardized antithrombotic protocol for acute flow diversion for ruptured IAs. There is an urgent need for more evidence-based guidelines to optimize antithrombotic treatment after flow diversion in the setting of subarachnoid hemorrhage.

**Supplementary Information:**

The online version contains supplementary material available at 10.1007/s00701-024-06029-7.

## Introduction

Simple coiling is the conventional endovascular care of ruptured intracranial aneurysms (IAs) [1–3]. However, for specific types of aneurysms, such as wide-necked aneurysms, dissecting aneurysms, blister-like aneurysms, and fusiform aneurysms, it is challenging to effectively secure the aneurysm with simple coiling [1, 2, 4–6]. Flow diverters have emerged as an effective solution to treat unruptured aneurysms, even with complex characteristics [7–9]. However, the use of flow diversion in acutely ruptured aneurysms is problematic because the aneurysm does not immediately occlude and to avoid severe thromboembolic complications, such as in-stent thrombosis and brain infarction, dual-antiplatelet therapy (DAPT) is strongly recommended [10–12]. Nevertheless, off-label use of flow diversion in this setting is becoming more frequent [2, 3, 10, 13].

Naturally, DAPT treatment during the acute phases of subarachnoid hemorrhage (SAH) predisposes to hemorrhagic complications. These include aneurysm re-rupture, hemorrhagic complications during placement and/or removal of external ventricular drains (EVD) and shunts, as well as extracranial hemorrhagic complications [2, 10]. Thus, careful consideration must be given to finetuning hemostasis when using flow diversion in ruptured IAs.

Most evidence regarding antiplatelet therapy after stenting comes from studies on acute coronary syndromes, which cannot be directly generalized to the cerebrovascular system, and especially not to aneurysmal SAH, where aneurysm rebleeding is a major concern [14, 15]. In case of flow diverter stent use in SAH, the most commonly used DAPT regimes are combinations of aspirin with clopidogrel, prasugrel, or ticagrelor [12, 13, 15]. Furthermore, after acute flow diverter stent deployment, additional use of a glycoprotein IIb/IIIa inhibitors, e.g., abciximab, eptifibatide, or tirofiban is frequent [10, 12, 13]. Still, current practices are not evidence based and the lack of evidence leads to significant practice variations between and within centers [16, 17].

Due to the lack of evidence-based guidelines for the antithrombotic treatment regime after acute flow diversion in the setting of SAH, we implemented a standardized antithrombotic treatment protocol in June 2021. In this study, we aimed to compare differences in treatment-related ischemic and hemorrhagic complications before and after the implementation of the standardized treatment protocol. We further reported patient outcome and aneurysm occlusion rates.

## Methods

### Study setting and patients

We conducted a retrospective study, including all consecutive patients with ruptured IAs treated in the acute setting with a flow diverter stent in Helsinki University Hospital, from September 2015 to February 2023. Helsinki University Hospital is the only neurosurgical unit covering the treatment of approximately 2.2 million inhabitants in Southern Finland. We did not include patients with acutely ruptured IAs that were treated without a flow diverter stent. A part of study population was included in a previous nationwide study [1].

### Antiplatelet treatment regime

The standardized antithrombotic treatment regime in the case of acute flow diversion after SAH was implemented in June 2021 (Supplementary Fig. 1). Briefly, the threshold for inserting an EVD was lowered if a flow diverter stent was going to be used. During the endovascular procedure, following the flow diverter stent deployment, aspirin was given followed by an eptifibatide bolus and infusion. Following the eptifibatide infusion, either prasugrel and aspirin were started as oral antiplatelet medications or if the patient remained intubated, cangrelor infusion was started in combination with aspirin. Daily assessments of conversion from cangrelor to prasugrel were made. Prophylactic low-molecular weight heparin was started on the third post-intervention day. In the acute phase, platelet function testing was not done due to the unreliability of the platelet function tests in the setting of SAH [18]. Prasugrel was generally continued for 6 months and aspirin for life.

### Neurointerventional treatment

A multidisciplinary team consisting of neurovascular surgeons and neurointerventionalists decided on the treatment strategy. The decision to use a flow diverter in the setting of IA rupture and acute SAH was considered as a last resort if other modalities were deemed unfeasable. The interventions were performed under general anesthesia through femoral or radial access using a 6–8F long sheath with or without a distal access catheter. An appropriate microcatheter was used for the selected flow diverter stent. In case of additional coiling, the coiling microcatheter was jailed in the aneurysm before stent placement. Stent simulation software was used at the discretion of the neurointerventionalist. All procedures were performed under heparinization targeting ACT values approximately two times higher than baseline.

### Definition of ischemic and hemorrhagic complications

We defined an ischemic complication as any new ischemic changes seen on post-interventional head CT, compared to the pre-interventional head CT, until the time of discharge from the neurosurgical ward. We classified ischemic complications as follows: major-stent related, minor-stent related, and delayed cerebral ischemia (DCI) related. MRIs were not regularly performed for all patients. If an ischemic lesion was noted on the MRI, it also had to be visible on the CT in order to be noted. We defined a hemorrhagic complication as an increase in blood in the brain parenchyma or subarachnoid space (ICH) or in the intraventricular space (IVH) not attributable to blood redistribution. We classified hemorrhagic complications as follows: major ICH/IVH, minor ICH/IVH, and aneurysm re-rupture. All images and complications were classified by a specialist in neurosurgery and interventional neuroradiology (R.R.) and interventional neuroradiology and diagnostic neuroradiology (J.N.). Since there are no standardized classifications regarding major and minor ischemic and hemorrhagic complications, this classification was based upon our clinical judgement and to increase transparency, all ischemic and hemorrhagic complications are displayed in Supplementary File 2.

### Clinical and radiological follow-up

From electronic health care records, we assessed the modified Rankin Scale (mRS) at 6 months based upon follow-up visits with neurosurgeons, neurologists, and rehabilitation physicians. We dichotomized the functional outcome to favorable (mRS 0–2) and unfavorable (mRS 3–6) [19]. If the patient died before the 6-month follow-up time, we noted the date of death. Radiological follow-ups for the aneurysms were carried out using DSA at 3–6 months and 12–24 months after the intervention, tailored to the patients’ needs.

### Data collection and variable definitions

We collected patient data, treatment data, and medication data from the hospital’s electronic health care records and prescription records. We obtained radiological images from the hospital picture archiving and communication system (PACS). We defined clinical SAH severity according to the World Federation of Neurosurgical Societies (WFNS) grading system upon admission [20]. We defined radiological SAH severity according to the modified Fisher grading scale [21]. DCI was diagnosed clinically whenever possible, based on criteria defined as (a) a new focal neurologic deficit or (b) a decrease in GCS ≥ 2 for at least 1 h, not ascribable to alternative diagnoses [22]. If reliable neurological assessment was not possible, DCI was diagnosed if there was severe radiological vasospasm. Moreover, if a patient developed new CT hypodensities outside of the direct vicinity of a previous focal lesion, it was considered a sign of DCI and treatment was initiated.

### Statistical analysis

Categorical variables are presented as numbers and percentages. Continuous variables were tested for normality using the Shapiro–Wilk test. Normally distributed variables are presented as means with standard deviations (SD) and non-parametric variables are presented as medians with interquartile ranges (IQR). Due to the small sample size, between-group comparisons were only done for the major outcomes. Categorical variables were compared using a Fisher’s exact test.

We divided patients into two time-based groups: those treated before the implementation of the standardized treatment-protocol (pre-group, treated before June 2021) and those treated after the implementation of the standardized treatment-protocol (post-group, treated after June 2021). We further divided the patients into groups based on actual received medication (treated according to protocol), as some of the patients in the pre-group might have been treated similarly to those in the post-group.

To assess the association between antithrombotic treatment strategy and outcome (mRS 0–2 vs. 3–6, alive or not at 6 months), we used logistic regression analysis, adjusting for age, sex, posterior circulation aneurysm, WFNS I–III vs. IV–V, and modified Fisher I–II vs. III–IV [23].

### Ethical aspect

Due to the single-center retrospective design of the study, informed consent was not required according to Finnish legislation. The study was approved by the local IRB.

## Results

Patient baseline characteristics are summarized in Table 1. Briefly, 39 patients (62% female) with 40 ruptured intracranial aneurysms were included in the study. The mean age was 55 (SD 13) years, half of the patients had a diagnosis of hypertension, one-fourth were active smokers, and one-fourth were on some form of antithrombotic medication prior to the SAH. Of all patients, 27 (69%) were treated in the pre-protocol era and 12 (31%) were treated in the post-protocol era. Most aneurysms were non-saccular (92%) and located in the posterior circulation (63%). The proportion of non-saccular (dissecting, blister, and fusiform) aneurysms were similar between the groups. Forty-four percent were WFNS grade of IV–V on admission. There were somewhat more poor grade SAH (WFNS IV–V) patients in the pre-group (48% vs. 33%) with more severe SAH (Fisher III–IV, 93% vs. 62%) than in the post-group. A median of 7 (IQR 5–9) CTs and a median of 0 (IQR 0–1) MRIs per patient were performed during the hospitalization.

**Table 1:** Patient baseline characteristics

Variable	All patients (*n* = 39)	Pre-group (*n* = 27)	Post-group (*n* = 12)
Age (mean, SD)	55 (13)	54 (12)	56 (14)
Female sex	24 (62%)	15 (56%)	9 (75%)
Hypertension	20 (51%)	12 (44%)	8 (67%)
Admission thrombocyte level (mean, SD)	242 (54)	241 (55)	243 (53)
Admission INR level (median, IQR)	1 (0.2)	1 (0.2)	1 (0.6)
Regular antithrombotic treatment prior to SAH			
No	29 (74%)	20 (74%)	9 (75%)
Antiplatelet	2 (5%)	1 (4%)	1 (8%)
Anticoagulation	8 (21%)	6 (22%)	2 (17%)
Smoking status			
No	19 (49%)	13 (48%)	6 (50%)
Active smoker	10 (26%)	8 (30%)	2 (17%)
Ex-smoker (> 6 mo)	5 (13%)	4 (15%)	1 (8%)
No data	5 (13%)	2 (7%)	3 (25%)
Aneurysm location			
Anterior circulation	15 (38%)	8 (30%)	7 (54%)
Posterior circulation	25 (63%)	19 (70%)	6 (46%)
Type of aneurysm			
Saccular	3 (8%)	2 (7%)	1 (8%)
Fusiform	6 (15%)	4 (15%)	2 (15%)
Blister	7 (18%)	5 (19%)	2 (15%)
Dissecting	24 (60%)	16 (60%)	8 (62%)
Aneurysm size (mm)			
Neck (median, IQR)	3 (2)	5 (4)	6 (2)
Height (median, IQR)	3 (4)	3 (4)	3 (5)
Dome (median, IQR)	2 (2)	2 (1)	3 (0)
WFNS SAH grade			
I–III	22 (56%)	14 (52%)	8 (67%)
IV–V	17 (44%)	13 (48%)	4 (33%)
Modified Fisher grade			
I–II	7 (18%)	2 (7%)	5 (42%)
III–IV	32 (82%)	25 (93%)	7 (58%)

Abbreviations: *WFNS*, World Federation of Neurological Surgeons; *SAH*, subarachnoid hemorrhage; *SD*, standard deviation; *IQR*, interquartile range

### Flow diverter treatment

The median time from SAH to flow diversion treatment was 1 day (IQR 0–1). In nine cases (23%), more than one flow diverter stents were used and in 18 cases (46%), additional coiling was performed (Table 2). In two patients, an additional flow diverter stent was deployed in another session due to re-rupture of the target aneurysm. The used stent models and their manufacturers are presented in Supplementary Table 1.

**Table 2:** Treatment characteristics

Variable	All patients (*n* = 39)	Pre-group (*n* = 27)	Post-group (*n* = 12)
Neurointerventional characteristics			
Flow diverter*	***n* = 53**	***n* = 37**	***n* = 16**
Surpass	20 (38%)	11 (30%)	9 (56%)
Fred	8 (15%)	7 (19%)	1 (6%)
Pipeline	24 (45%)	18 (49%)	6 (38%)
Silk	1 (2%)	1 (3%)	0
More than one flow diverter stent used	9 (23%)	7 (26%)	2 (17%)
Additional coiling	18 (46%)	11 (41%)	7 (58%)
Antithrombotic medication treatment characteristics			
Glycoprotein IIb/IIIa receptor antagonist bolus and/or infusion†	25 (64%)	13 (48%)	12 (100%)
Immediate P2Y_12_ receptor inhibitor loading dose†	24 (62%)	13 (48%)	11 (92%)
Postprocedural antithrombotic therapy			
Aspirin + clopidogrel	2 (5%)	2 (7%)	0
Aspirin + tinzaparin	13 (33%)	13 (48%)	0
Aspirin + prasugrel**†**	18 (46%)	10 (37%)	8 (67%)
Prasugrel + tinzaparin§	2 (5%)	2 (7%)	0
Aspirin + cangrelor**†**	3 (8%)	0	3 (25%)
None‡	1 (3%)	0	1 (8%)
Intensive care characteristics and complications			
External ventricular drain			
Before neurointervention	29 (74%)	19 (70%)	10 (83%)
After neurointervention	3 (8%)	3 (11%)	0
Not at all	7 (18%)	5 (19%)	2 (17%)
Occlusion of EVD	7 (18%)	5 (19%)	2 (17%)
Revision of EVD	5 (13%)	4 (15%)	1 (8%)
Shunt surgery	15 (38%)	11 (41%)	4 (33%)
Shunt revision surgery	5 (13%)	4 (15%)	1 (8%)
DVT/PE	4 (10%)	2 (7%)	2 (17%)
Delayed cerebral ischemia	16 (41%)	12 (44%)	4 (33%)
Length of stay, days (median, IQR)			
Intensive care unit	12 (13)	13 (14)	9 (10)
University hospital	17 (18)	21 (19)	13 (11)

^*^Total number of stents used

^†^Considered to be treated according to protocol

^‡^One patient had a massive intra-interventional bleed and did not get any post-procedural antithrombotic medications

^§^Early tinzaparin started directly after the intervention

Abbreviations: *EVD*, external ventricular drain; *DVT*, deep vein thrombosis; *PE*, pulmonary embolism; *IQR*, interquartile range

### Antithrombotic treatment

Differences in antithrombotic medication treatment strategies between the pre-protocol and post-protocol groups are shown in Table 2. In the pre-protocol group, immediate glycoprotein IIb/IIIa inhibitor bolus and/or infusion was given in 48% of cases compared to 100% in the post-protocol group. An immediate P2Y12 receptor inhibitor loading dose was given to 48% of patients in the pre-protocol group compared to 92% in the post-protocol group. Early DAPT was given to 44% of patients in the pre-group compared to 92% in the post-group. In the pre-protocol group, the most frequent postprocedural antithrombotic medication regime was aspirin + tinzaparin (48%), followed by aspirin + prasugrel (37%). In comparison, in the post-protocol group, the most frequent early postprocedural antithrombotic medication regime was aspirin + prasugrel (67%) and aspirin + cangrelor (25%).

### Intensive care treatment and complications

Eighty-two percent of patients received an EVD during the intensive care stay, mostly before the stenting procedure (74%). There were no notable differences in rates of occluded EVDs, need for EVD revisions, need for shunt surgery, incidence of deep venous thrombosis or pulmonary embolisms, or DCI between the pre-protocol and post-protocol groups (Table 2).

### Ischemic and hemorrhagic complications

Ischemic complications and hemorrhagic complications are summarized in Table 3 and shown in Supplementary File 2.

**Table 3:** Complications and outcomes

Variable	All patients (*n* = 39)	Pre-group (*n* = 27)	Post-group (*n* = 12)	*p*-value
Ischemic complications	15 (38%)	10 (37%)	5 (42%)	0.99
Major stent-related	10 (26%)	7 (26%)	3 (25%)	
Minor stent-related	3 (8%)	2 (7%)	1 (8%)	
DCI-related	2 (5%)	1 (4%)	1 (8%)	
Hemorrhagic complications	12 (31%)	8 (30%)	4 (33%)	0.99
Major ICH/IVH	5 (13%)	3 (11%)	2 (17%)	
Minor ICH/IVH	4 (10%)	2 (7%)	2 (17%)	
Aneurysm re-rupture	3 (8%)	3 (11%)	0 (0%)	
Aneurysm occlusion*				0.53
No filling or entry remnant (< 5%)	25/28 (89%)	16/19 (84%)	9/9 (100%)	
Subtotal filling or total filling	3/28 (11%)	3/19 (16%)	0/0 (0%)	
Modified Rankin Scale at 6 months†				0.99
mRS 0–2	22 (56%)	15 (56%)	7 (58%)	
mRS 3–6	17 (44%)	12 (44%)	5 (42%)	
Death within 6 months	12 (31%)	8 (30%)	4 (33%)	0.99

^*^Twenty-eight out of 39 patients underwent an angiographic control after a median of 5 (interquartile range 3–7) months

^†^The modified Rankin Scale was assessed after a median of 7 (interquartile range 6–12) months

Abbreviations: *ICH*, intracerebral hemorrhage; *IVH*, intraventricular hemorrhage; *mRS*, modified Rankin Scale

The overall incidence of ischemic complications was 38%, with no between-group difference (37% vs. 42%, *p* = 0.99). There were no between-group differences in the incidences of major stent-related, minor stent-related, or DCI-related ischemic lesions.

The overall incidence of hemorrhagic complications was 31%, with no between-group difference (30% vs. 33%, *p* = 0.99). Four of the 12 (33%) hemorrhagic complications were related to the EVD (three minor ICH, one major IVH). Furthermore, one major IVH occurred following intraventricular alteplase treatment and one minor IVH occurred following a shunt surgery 6 days earlier.

There were three aneurysm re-ruptures in the pre-group (all treated with aspirin and tinzaparin) compared zero re-ruptures in the post-group. The three re-ruptures occurred 6, 11, and 35 days after the index treatment.

When analyzing patients according to received treatment, patients treated according to protocol had more ischemic complications (50% vs. 26%, *p* = 0.20), but fewer hemorrhagic complications (15% vs. 47%, *p* = 0.038, Table 4). Still, there was no difference in the incidence of major stent-related ischemic complications between those treated according to protocol versus not (30% vs. 21%).

**Table 4:** Complications and outcomes according to received treatment

Variable	All patients (*n* = 39)	Treated according to protocol (*n* = 20)	Not treated according to protocol (*n* = 19)	*p*-value
Ischemic complications*	15 (38%)	10 (50%)	5 (26%)	0.20
Major stent-related	10 (26%)	6 (30%)	4 (21%)	
Minor stent-related	3 (8%)	3 (15%)	0 (0%)	
DCI-related	2 (5%)	1 (5%)	1 (5%)	
Hemorrhagic complications	12 (31%)	3 (15%)	9 (47%)	0.038
Major ICH/IVH	6 (15%)	1 (5%)	5 (26%)	
Minor ICH/IVH	3 (8%)	2 (10%)	1 (5%)	
Aneurysm re-rupture	3 (8%)	0 (0%)	3 (16%)	
Aneurysm occlusion*				0.54
No filling or entry remnant (< 5%)	25/28 (89%)	15/16 (94%)	9/11 (82%)	
Subtotal filling or total filling	3/28 (11%)	1/16 (6%)	2/11 (18%)	
Modified Rankin Scale at 6 months†				0.11
mRS 0–2	22 (56%)	14 (70%)	8 (42%)	
mRS 3–6	17 (44%)	6 (30%)	11 (58%)	
Death within 6 monhts	12 (31%)	4 (20%)	8 (42%)	0.31

^*^Twenty-eight out of 39 patients underwent an angiographic control after a median of 5 (IQR 3–6) months

^†^The modified Rankin Scale was assessed after a median of 6 (IQR 4–8) months

Abbreviations: *ICH*, intracerebral hemorrhage; *IVH*, intraventricular hemorrhage; *IQR*, interquartile range; *mRS*, modified Rankin Scale

### Angiographic outcome

Twenty-six out of 28 alive patients underwent a DSA control after a median of 5 (IQR 3–6) months. Of them, nine out of nine (100%) in the post-group had an adequately occluded aneurysm compared to 16 out of 19 (84%) in the pre-group (*p* = 0.53) (Table 3). Adequate occlusion rate was 94% in those actually treated according to protocol compared to 82% in those not treated according to protocol (*p* = 0.54) (Table 4).

### Clinical outcome

The median follow-up time was 6 months (IQR 4–8). There were no between-group differences in the rates of favorable functional outcome (56% vs. 58%, *p* = 0.99) or death within 6 months (30% vs. 33%, *p* = 0.99, Table 3). Patients treated according to protocol versus not treated according to protocol had a higher rate of favorable functional outcome (70% vs. 42%, *p* = 0.11) and fewer deaths within 6 months (20% vs. 42%, *p* = 0.31, Table 4).

## Discussion

### Key findings

In this retrospective single-center study, 38% of the patients had an ischemic complication and 31% had a hemorrhagic complication after an acute flow diversion of a ruptured intracranial aneurysm. Furthermore, a favorable functional outcome was achieved in 56% of patients and an adequate aneurysm occlusion in 89% of patients at 6-month follow-up. We found no notable differences among patients treated according to a new standardized antithrombotic medication protocol compared to those treated before the implementation of the protocol. Still, patients treated according to protocol had significantly fewer hemorrhagic complications.

### Flow diverters in acute subarachnoid hemorrhage

Our findings indicate that flow diversion is an effective treatment method for ruptured aneurysms, albeit with the high risk for ischemic and hemorrhagic complications. There were three patients with a re-rupture of the target aneurysm, all of which were treated with LMWH and aspirin, which might prevent the aneurysm from thrombosing [1]. Still, the overall aneurysm occlusion rate was 89%, which is in line with previous studies (76–100%) [24–27]. Furthermore, 56% of patients had a favorable functional outcome, which is comparable with SAH patients whose aneurysms were treated using other modalities [28]. Thus, although the rate of complications was high, our results suggest the clinical sequelae of the ischemic and hemorrhagic complications were not majorly disabling and patient outcome was acceptable considering the challenging nature of the aneurysms and the high clinical severity of the SAH.

### Hemorrhagic and ischemic complications

We found relatively high rates of ischemic and hemorrhagic complications, with no major differences between those treated according to protocol and not. A previous meta-analysis including 223 ruptured aneurysms reported an overall ischemic and hemorrhagic complication rate of 8% and 7%, respectively, but as high as 18%, and 27%, respectively, for posterior circulation aneurysms [23]. In comparison, the occurrence of flow diverter-related complications after treating an unruptured aneurysm is reported to be 5–22% [29, 30]. Our reported complications rates are higher than previously described (ischemic 38% and hemorrhagic 31%), partly due to the meticulous detection of minor complications without clinical sequela. Be it noted that 13% of the ischemic complications were not stent related and that even minor asymptomatic imaging positive ischemic and hemorrhagic lesions were included as complications (Supplementary File 2). Furthermore, probable reasons for the higher complications rate noted in our study are the higher rate of clinically poor grade SAH (44% vs. 27%), more severe SAH (modified Fisher III–IV 82% vs. 68%), the higher proportion of posterior circulation aneurysms (63% vs. 33%), and higher proportion of non-saccular (dissecting, fusiform, blister) aneurysms (92% vs. 81%) [23].

### Clinical outcome

We found a slightly lower mortality rate (20% vs. 42%) and lower rate of unfavorable functional outcome (30% vs. 58%) in the group treated according to the standardized protocol compared to those not treated according to the standardized protocol. However, the proportion of patients with a poor grade SAH was higher in the pre-protocol group, which may explain these differences (Supplementary Table 2). Previous reported mortality rates range from 4.5 to 19%, although some studies have reported only treatment-related mortality [23–25, 27].

### Choice of antiplatelet medication

There is a wide variation in the choice of P2Y12 antagonist for flow diversion [15, 31, 32]. In the implemented protocol, we did not choose ticagrelor because it is a reversible P2Y12 antagonist, whereas prasugrel and clopidogrel are irreversible P2Y12 antagonists. Thus, in the setting of a major hemorrhagic complication, the antiplatelet effect of prasugrel and clopidogrel is at least partially reversible with platelet transfusions. Additionally, clopidogrel has a complicated metabolism and is associated with resistance in up to 40% of people [33–36]. Thus, prasugrel seems to have the most favorable profile in the case of acute flow diversion after SAH. Noteworthy, the implemented protocol initiates the antithrombotic treatment after the deployment of the flow diverter and does not include the option of a pre-intervention loading of antiplatelet medication, as is employed when treating unruptured aneurysms with flow diversion*.* Thus, another treatment strategy could be to load the patients with antiplatelets before the intervention to allow for platelet inhibition before deployment of the flow diverter stent. Delayed flow diversion treatment with two or more days compared to within 2 days of ictus does not seem to affect the treatment risks, although the risk for rebleeding increases [37]. Yet, pre-intervention loading of antiplatelet drugs might potentially increase the risk of aneurysm rebleeding but should be explored as an alternative strategy considering the high rate of ischemic complications [37].

### Single antiplatelet therapy

The potential complication of antithrombotic medication in the setting of SAH is hemorrhagic complications. We found the overall incidence of hemorrhagic complications to be 31%, although only approximately half of these were deemed as major hemorrhagic complications. One way in the future to avoid hemorrhagic complications could be to substitute DAPT with single antiplatelet treatment (SAPT). Recently, flow diverter stents with surface modifications have been introduced and preliminary reports have not found any major differences in complications or outcomes between SAPT and DAPT after acute flow diversion after using only prasugrel or ticagrelor [27, 38]. Still, further studies are needed to assess whether the use of SAPT vs. DAPT is preferrable after flow diversion for acute SAH.

### Limitations

There are limitations to our study. Although we included consecutive patients, the retrospective nature of this study could insinuate bias. Moreover, the small cohort increases the risk for type I and II errors. Thus, the between-group comparisons should be interpreted with caution. Furthermore, this was a single-center study and results might not be applicable elsewhere.

## Conclusion

We found a relatively high rate of ischemic and hemorrhagic complications after acute flow diversion of ruptured IAs. We did not notice any major differences in the incidence of ischemic or hemorrhagic complications after the implementation of a standardized antithrombotic protocol for acute flow diversion for ruptured IAs. Considering the increasing use of off-label acute flow diversion after acutely ruptured IAs, there remains an urgent need for improved evidence in terms of antithrombotic medication strategies.

## Supplementary Information

Below is the link to the electronic supplementary material.

Supplementary file1 (PDF 69 KB)

Supplementary file2 (DOCX 14237 KB)

Supplementary file3 (DOCX 23 KB)

## Data Availability

The dataset analyzed in the study is not publicly available and we are unable to share the data for public use. Nevertheless, eligible researchers may seek permission to access the healthcare information the local institutional review board of Helsinki University Hospital and/or through the Finnish Social and Health Data Permit Authority at findata.fi/en/.
