# Supplementary figures and images for: Flow diversion of ruptured intracranial aneurysms: a single-center study with a standardized antithrombotic treatment protocol
Source: Acta Neurochir (Wien). 2024 Mar 11;166(1):130. doi: 10.1007/s00701-024-06029-7 (PMC10927838; doi:10.1007/s00701-024-06029-7)

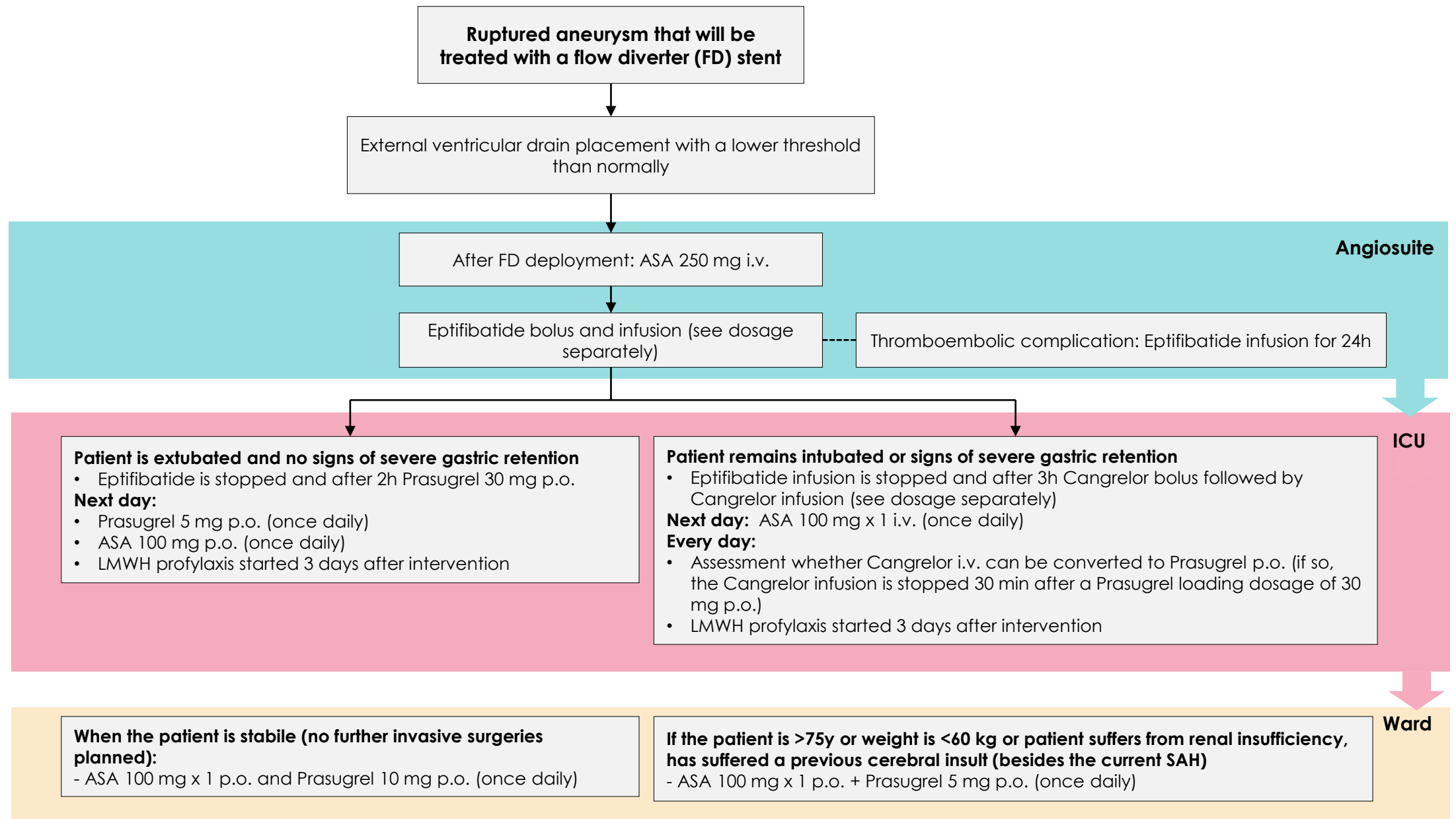

Supplement: Supplementary file 1 — Supplementary file1 (PDF 69 KB) [file 701_2024_6029_MOESM1_ESM.pdf]
