# Supplementary material for: Flow diversion of ruptured intracranial aneurysms: a single-center study with a standardized antithrombotic treatment protocol
Source: Acta Neurochir (Wien). 2024 Mar 11;166(1):130. doi: 10.1007/s00701-024-06029-7 (PMC10927838; doi:10.1007/s00701-024-06029-7)
Supplement: Supplementary file 2 — Supplementary file2 (DOCX 14237 KB) [file 701_2024_6029_MOESM2_ESM.docx]

# **Supplementary File**

*Supplementary file showing all hemorrhagic complications and ischemic complications. Image to the left represent the pre-complication image and the image to the right the complication. The location of the ruptured aneurysm is also noted.*

Aneurysm rebleed 1, vertebral artery aneurysm

| 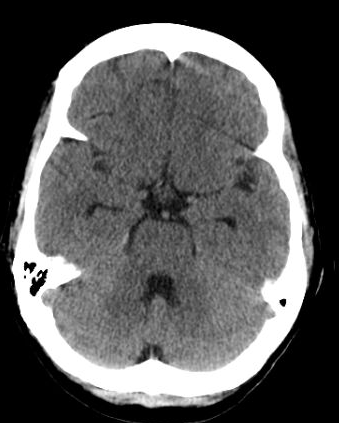 | 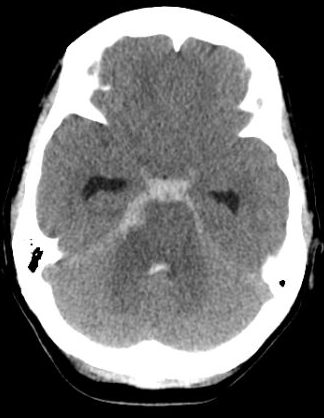 |
| --- | --- |

Aneurysm rebleed 2, vertebral artery aneurysm

| 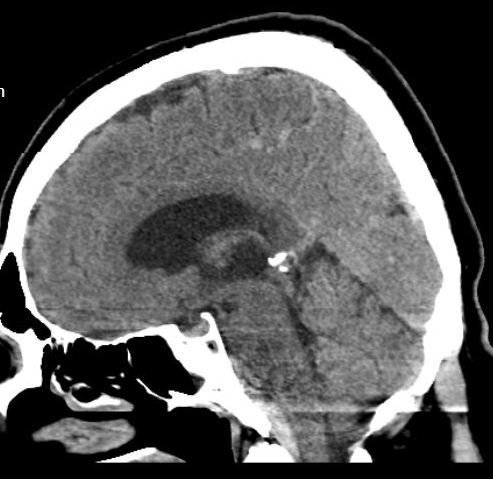 | 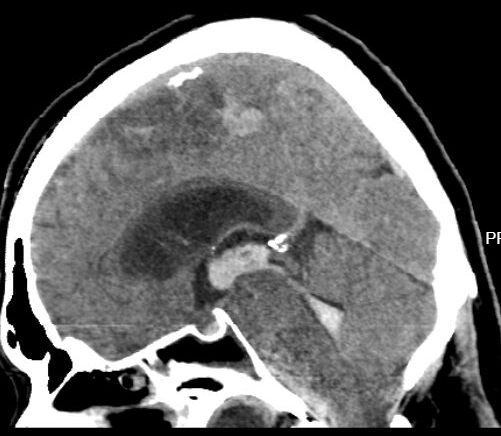 |
| --- | --- |

Aneurysm rebleed 3, ICA aneurysm

| 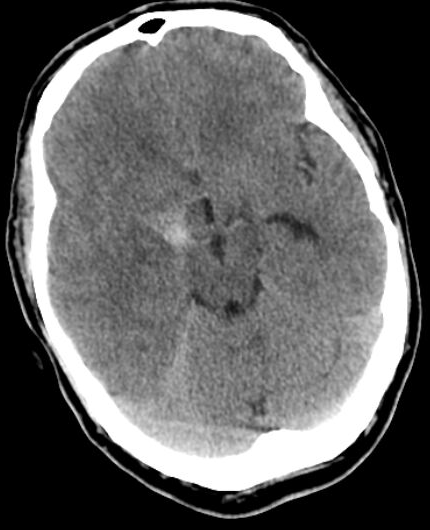 | 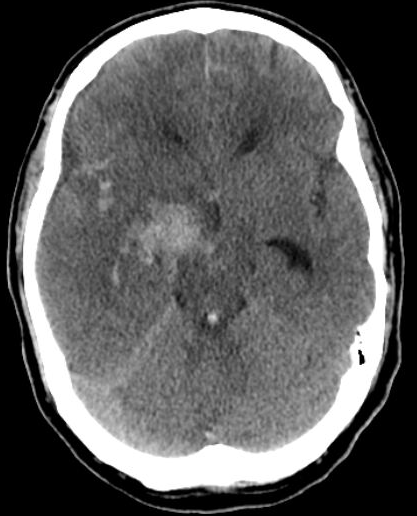 |
| --- | --- |

Major IVH 1, basilar artery aneurysm

| 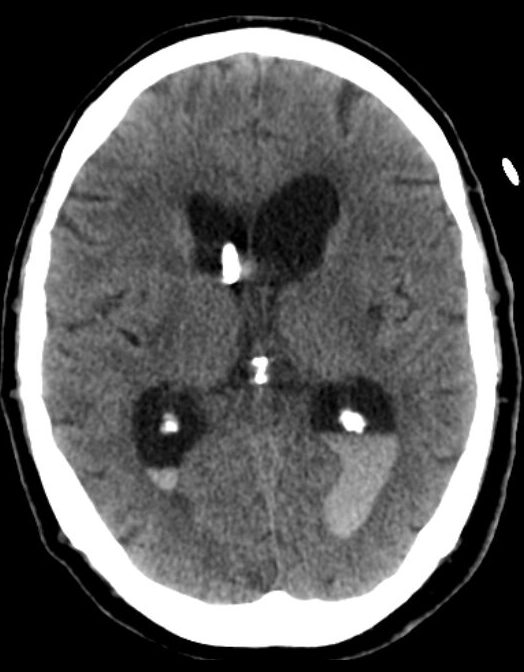 | 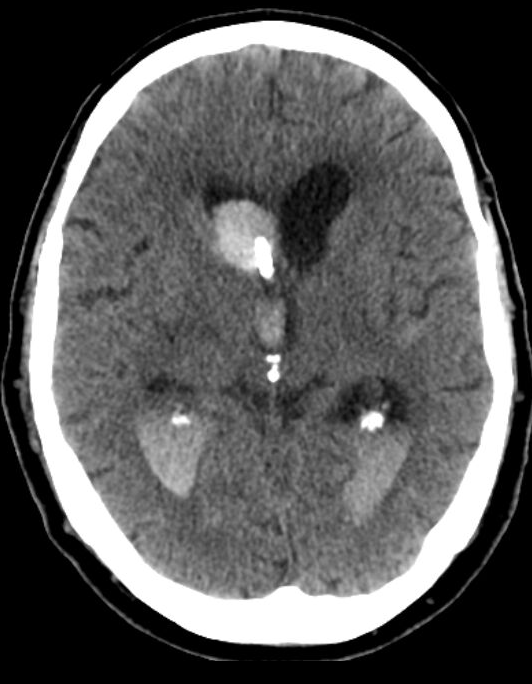 |
| --- | --- |

Major IVH 2, basilar artery aneurysm

| 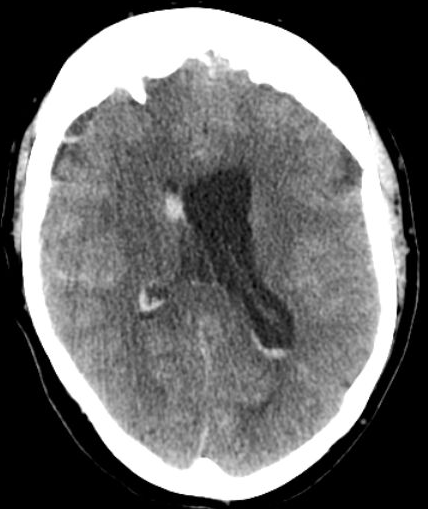 | 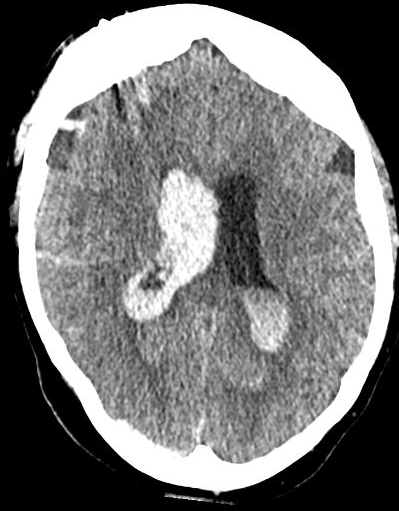 |
| --- | --- |

Minor IVH 1, vertebral artery aneurysm

| 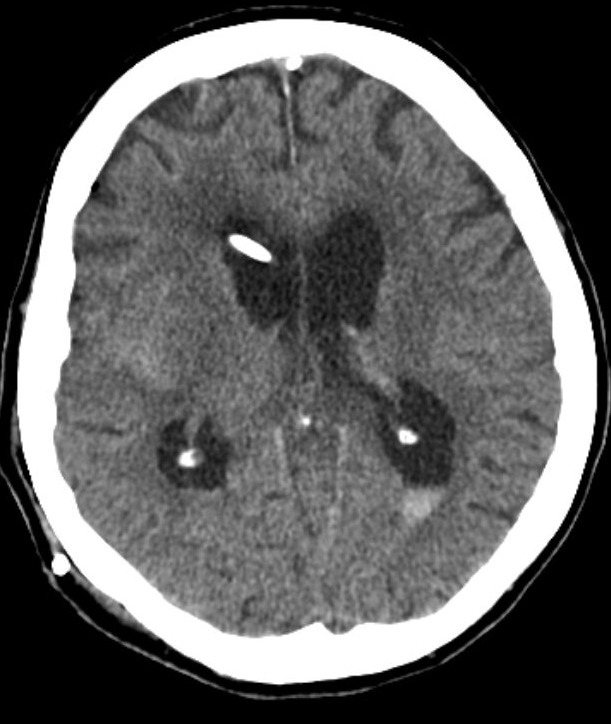 | 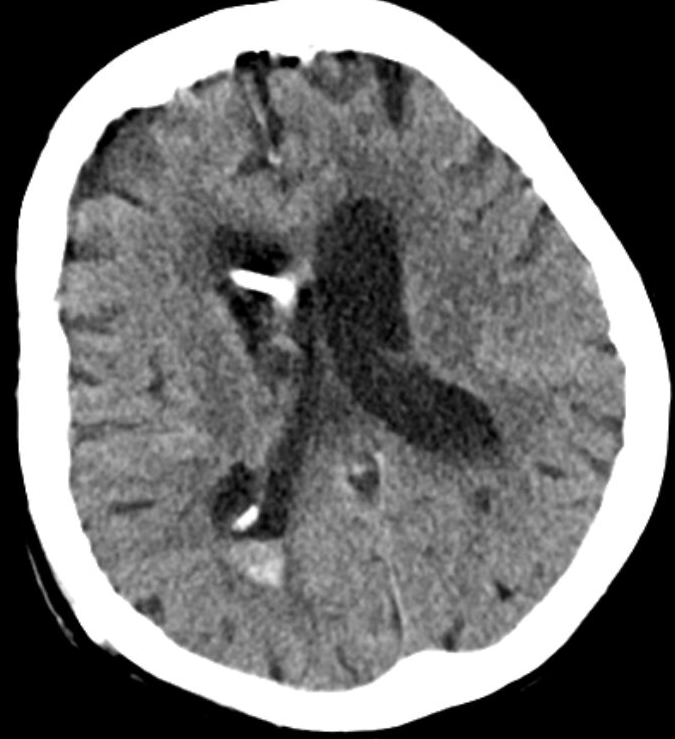 |
| --- | --- |

Major ICH 1, posterior cerebral artery aneurysm

| 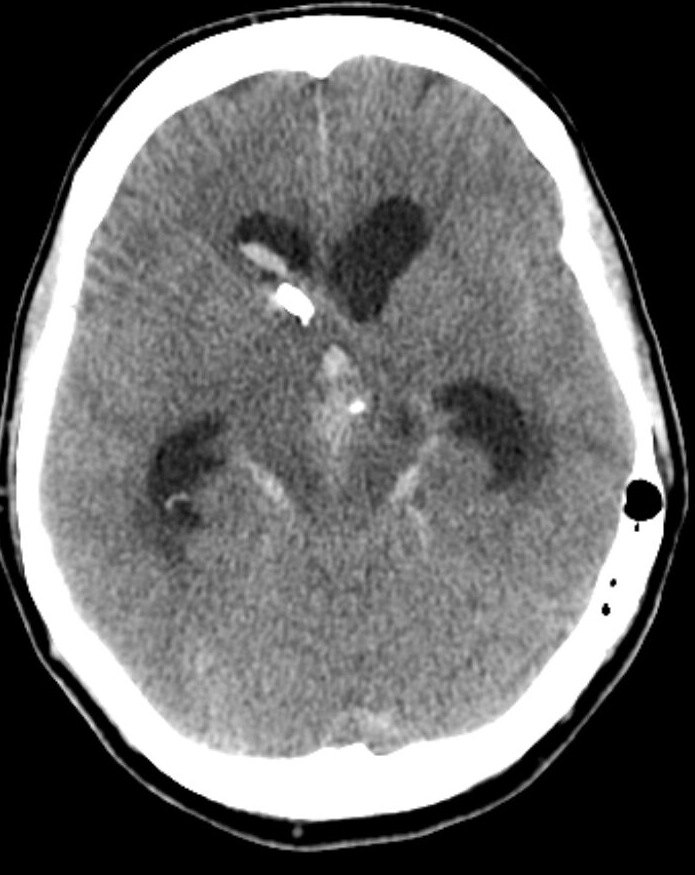 | 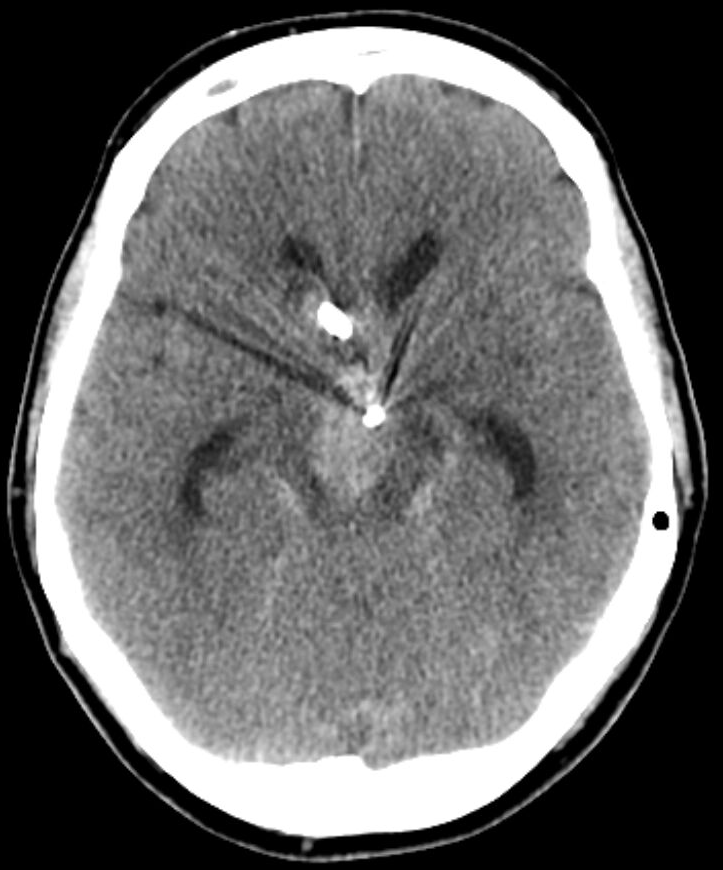 |
| --- | --- |

Major ICH 2, ICA aneurysm

| 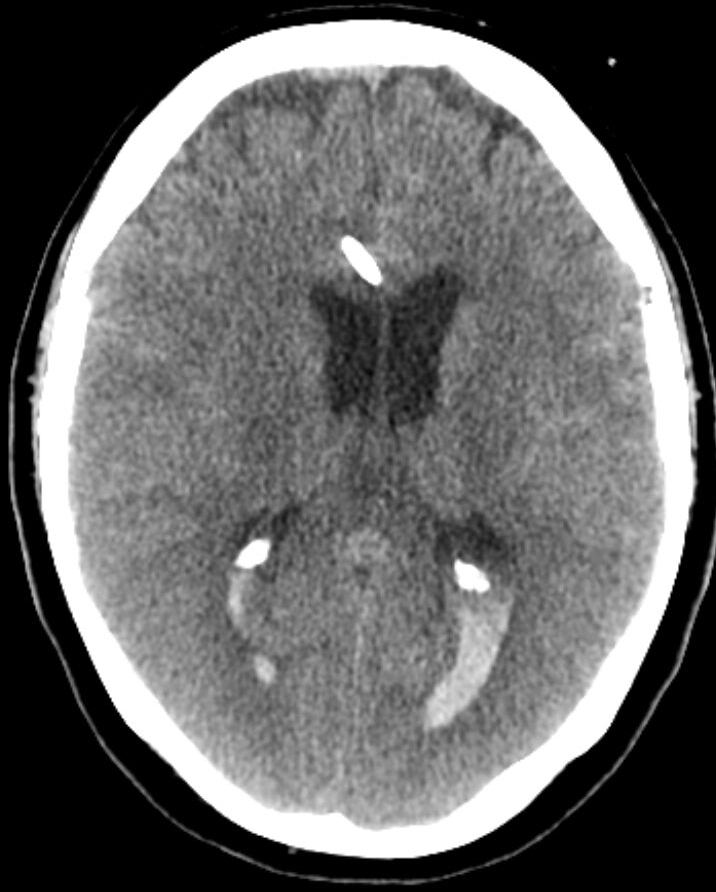 | 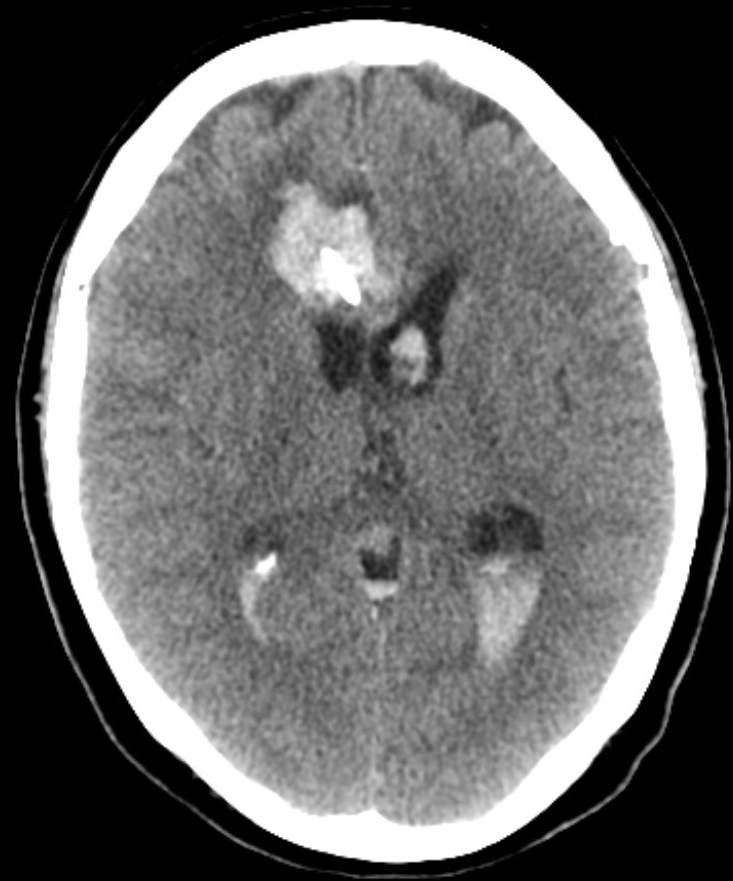 |
| --- | --- |

Major ICH 3, ICA aneurysm

| 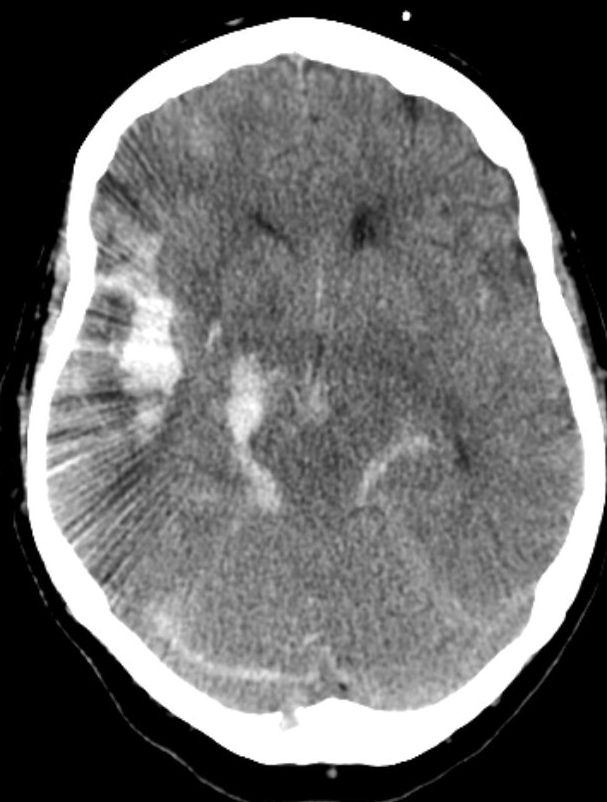 | 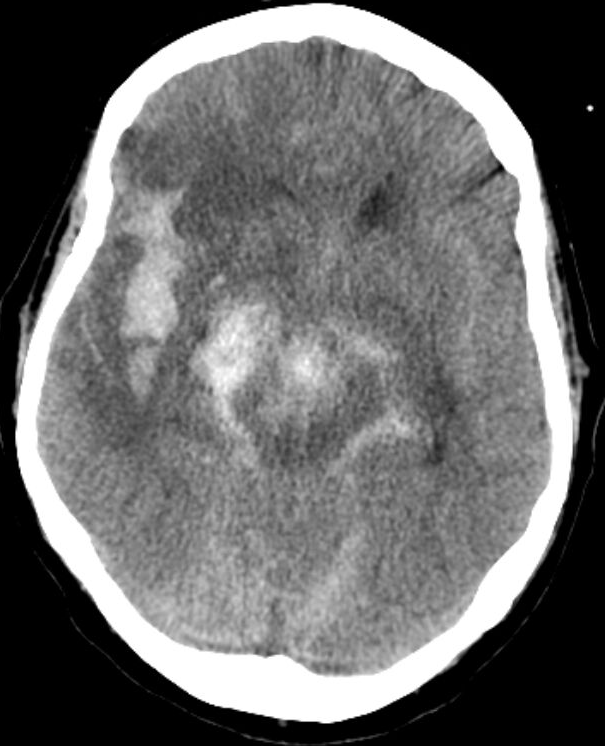 |
| --- | --- |

Minor ICH 1, ICA aneurysm

| 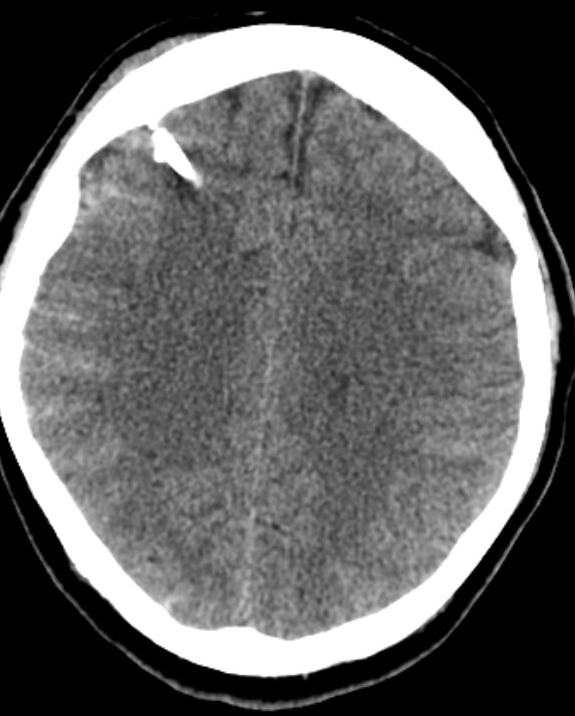 | 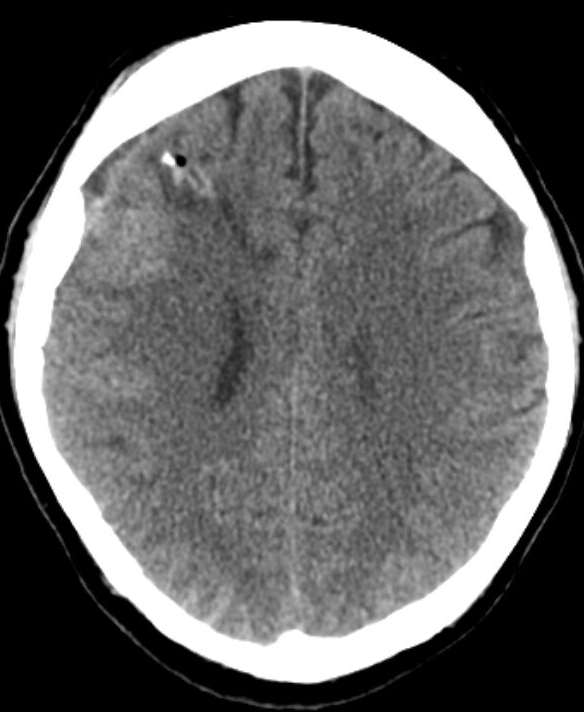 |
| --- | --- |

Minor ICH 2, basilar artery aneurysm

| 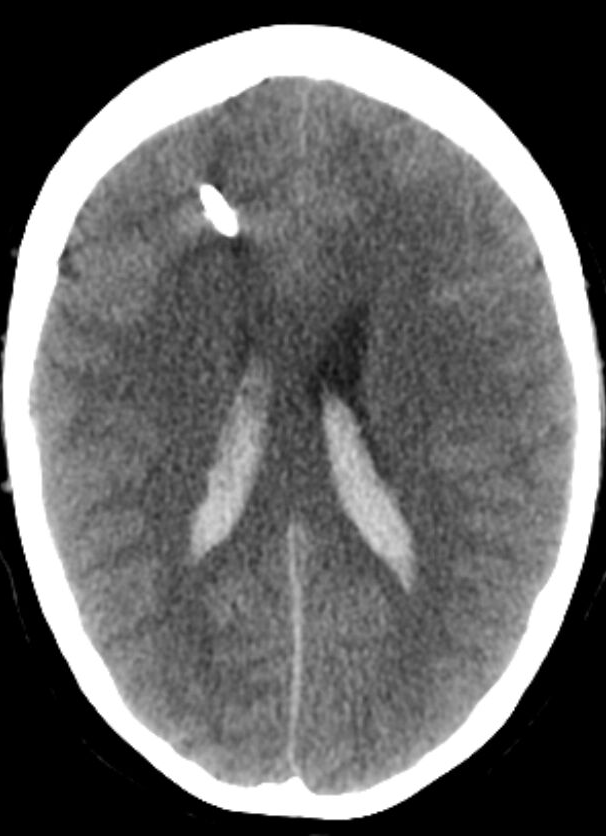 | 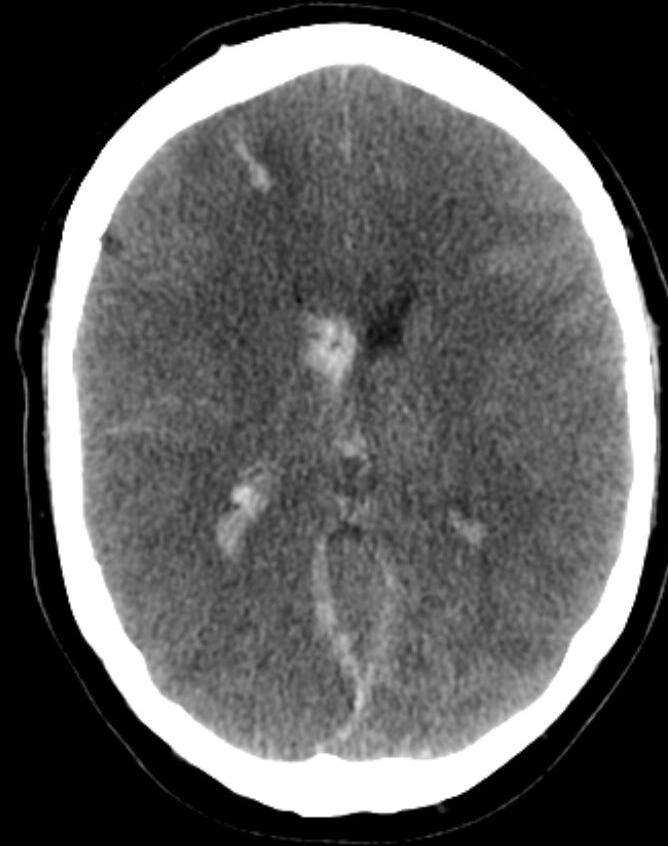 |
| --- | --- |

Minor ICH 3, ICA aneurysm

| 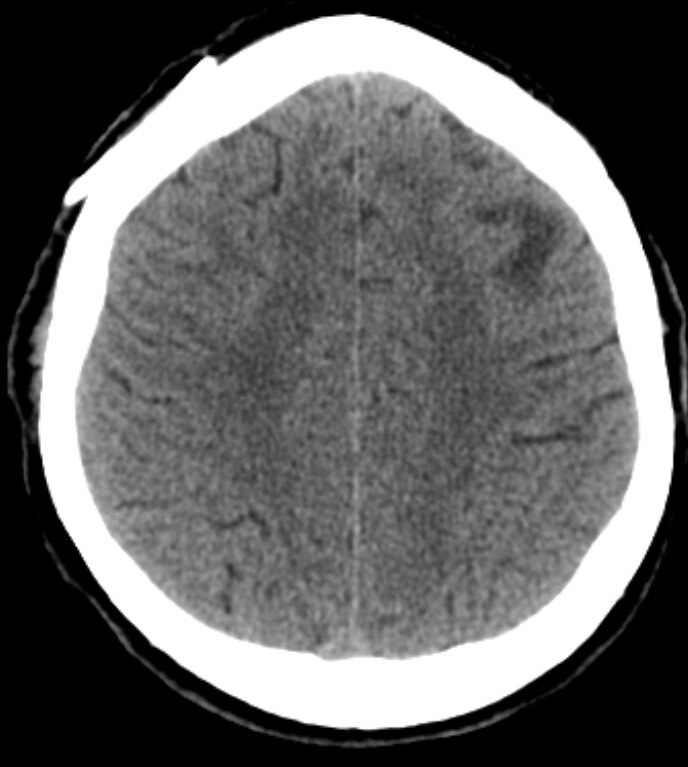 | 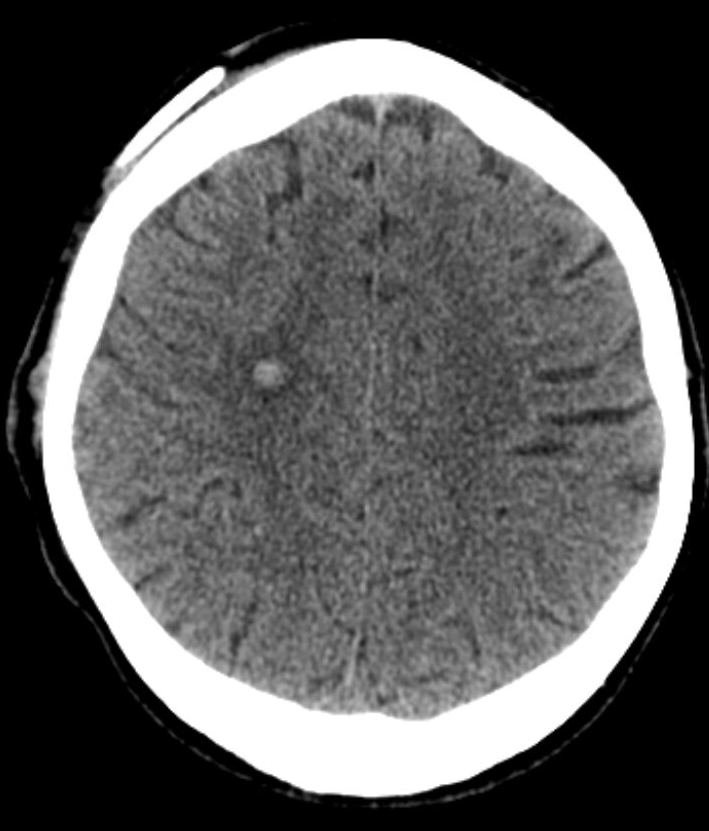 |
| --- | --- |

Major ischemic complication – Not stent related 1, pericallosal aneurysm

| 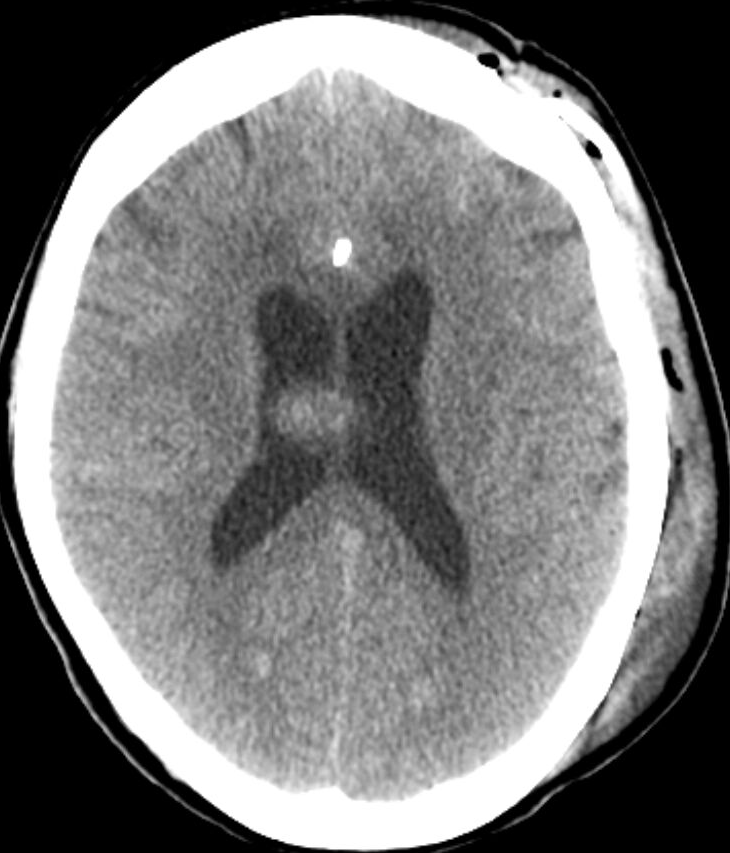 | 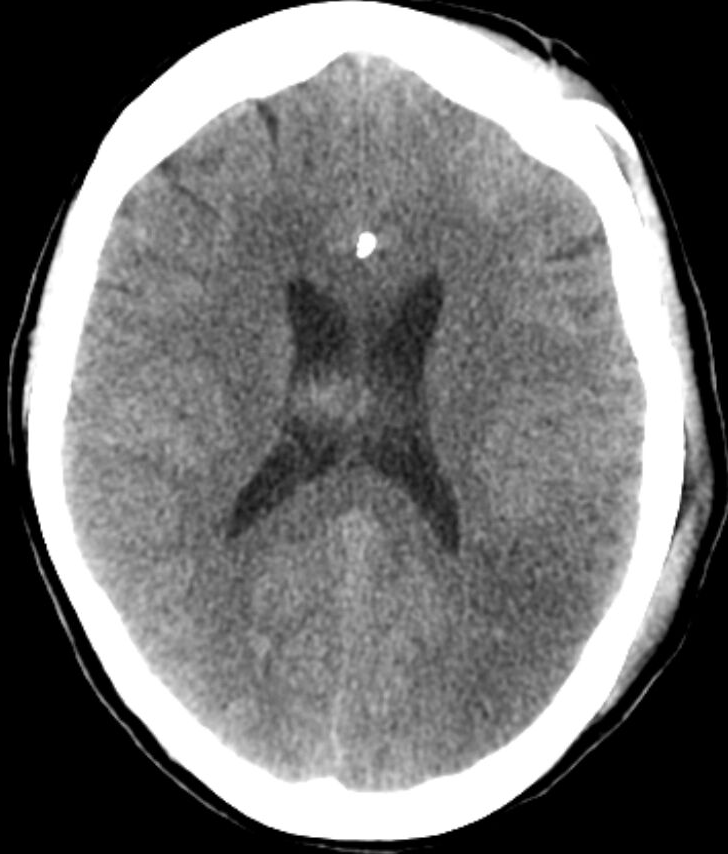 |
| --- | --- |

Major ischemic complication – Not stent related 2, ICA aneurysm

| 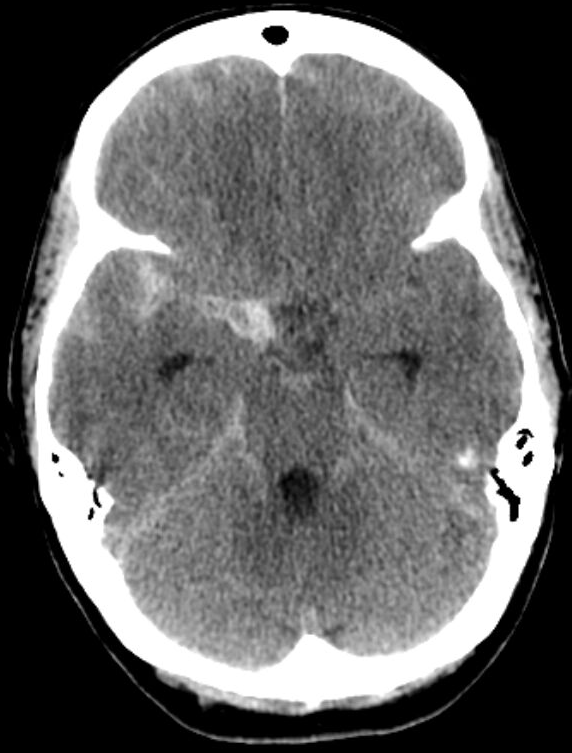 | 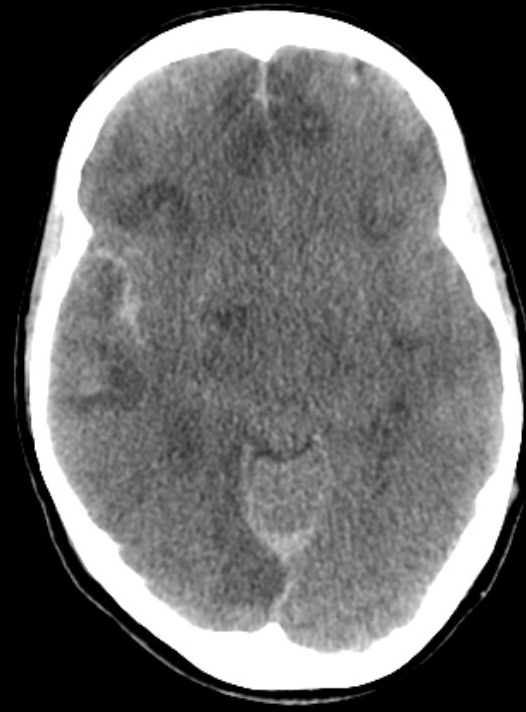 |
| --- | --- |

Major ischemic complication – Stent related 1, basilar artery aneurysm. *MRI showed for visualization of the brain stem ischemia*.

| 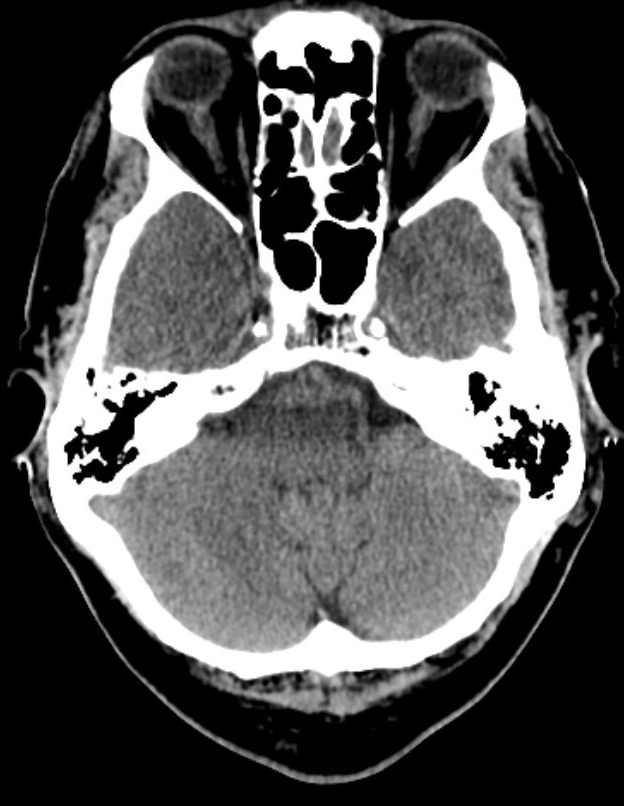 | 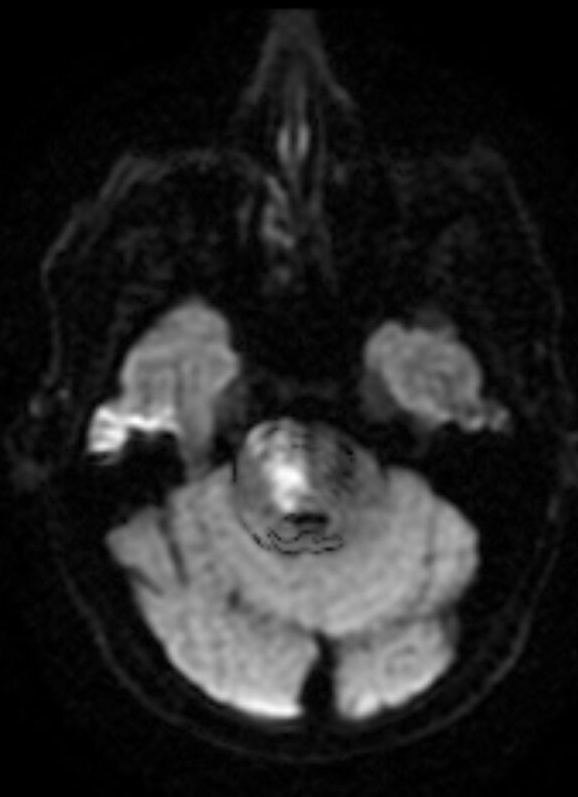 |
| --- | --- |

Major ischemic complication – Stent related 2, basilar artery aneurysm

| 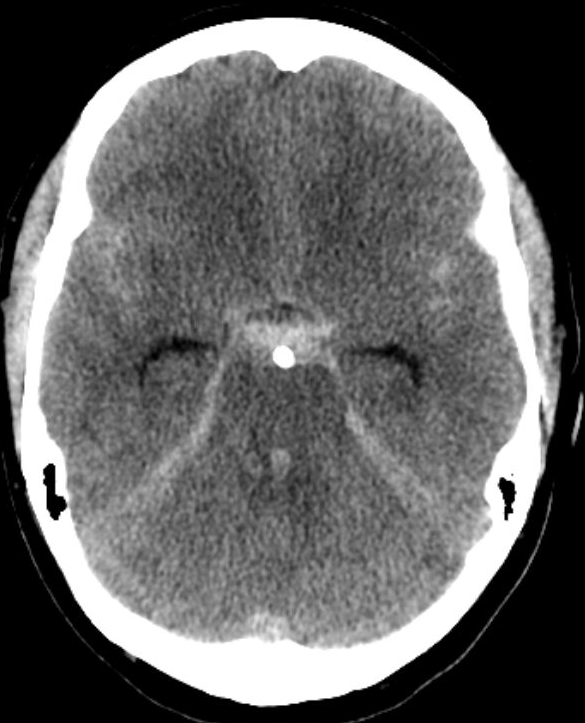 | 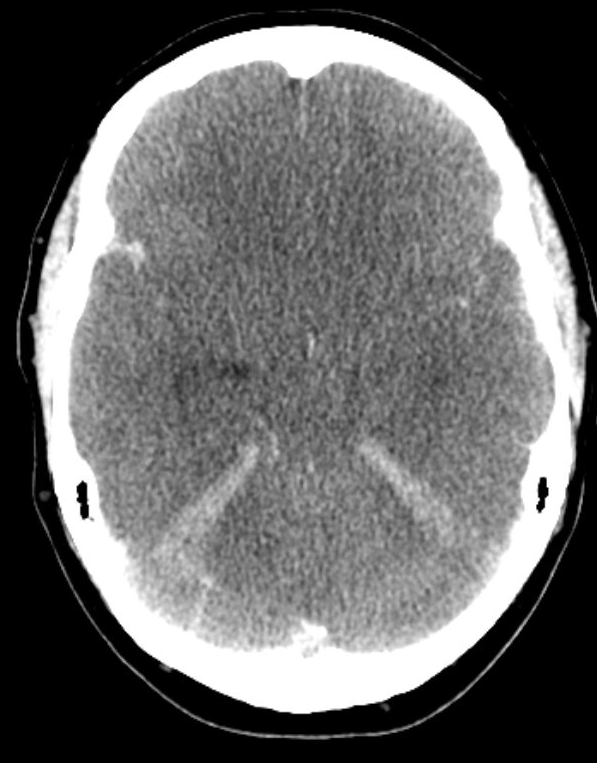 |
| --- | --- |

Major ischemic complication – Stent related 3, ICA aneurysm

| 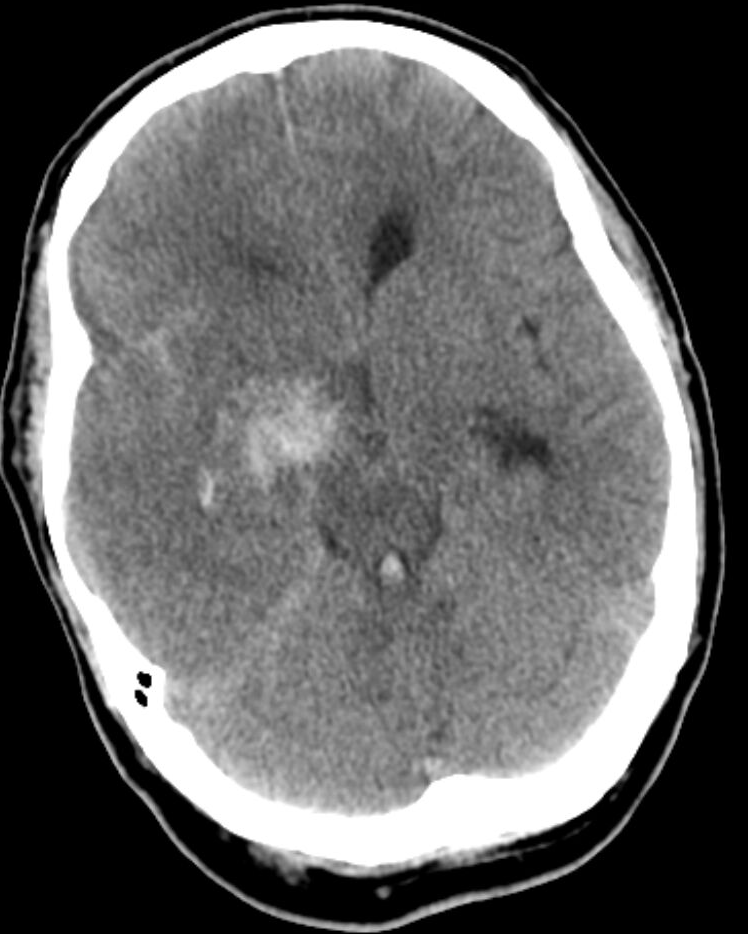 | 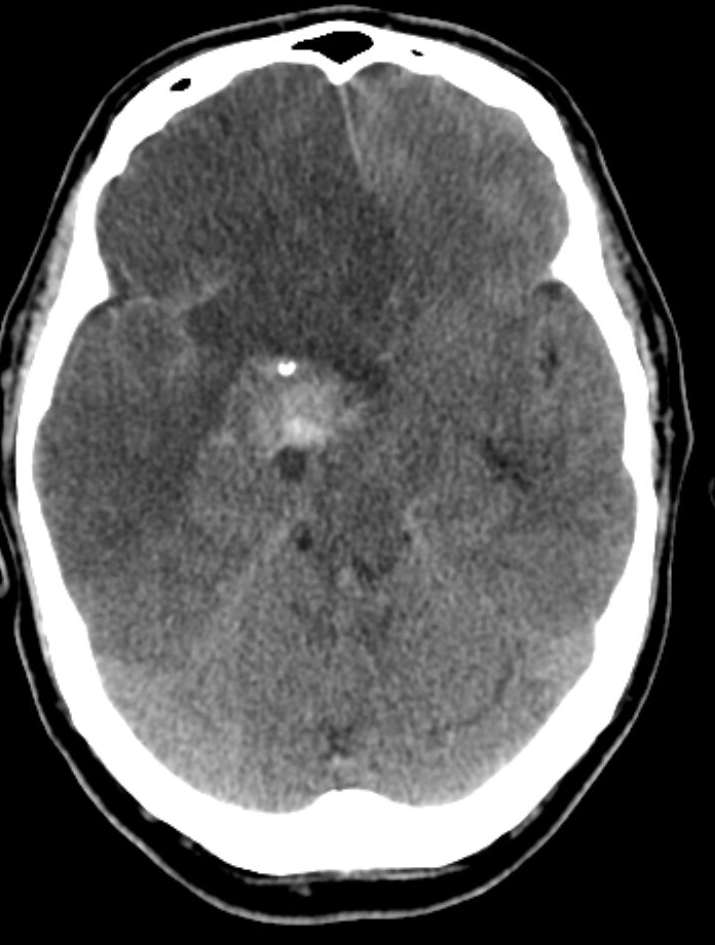 |
| --- | --- |

Major ischemic complication – Stent related 4, vertebral artery aneurysm

| 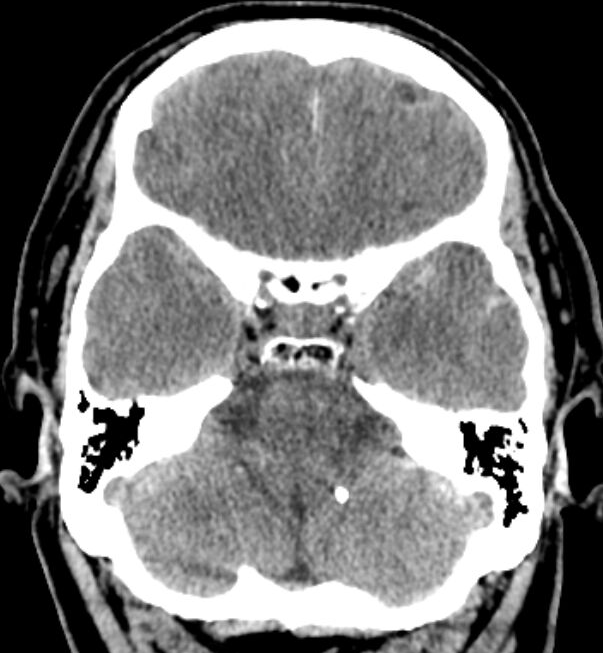 | 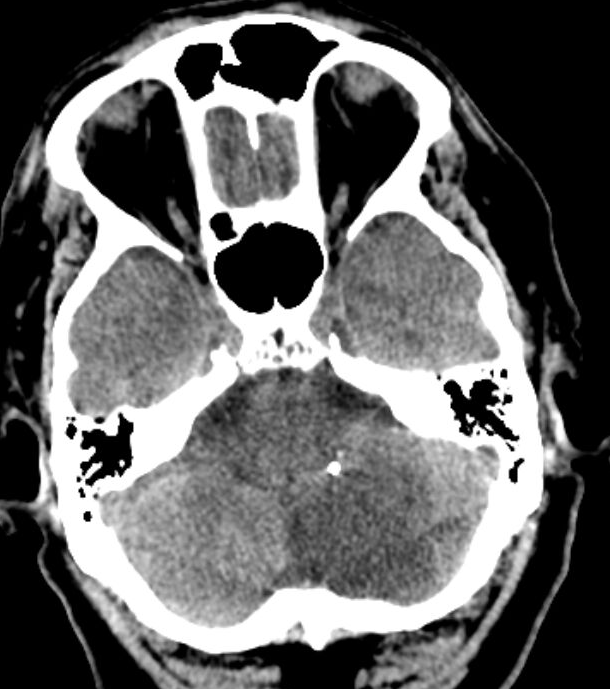 |
| --- | --- |

Major ischemic complication – Stent related 5, posterior cerebral artery aneurysm

| 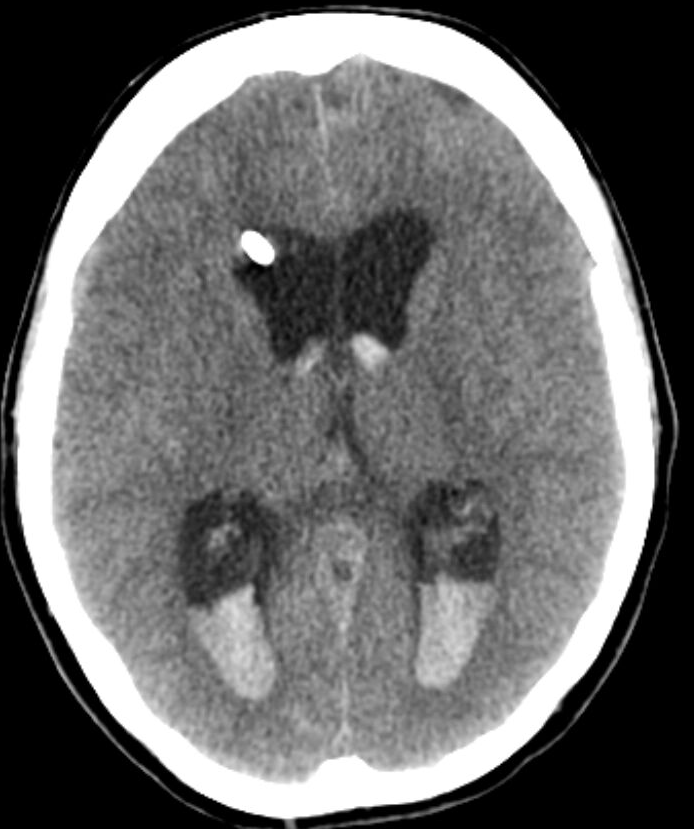 | 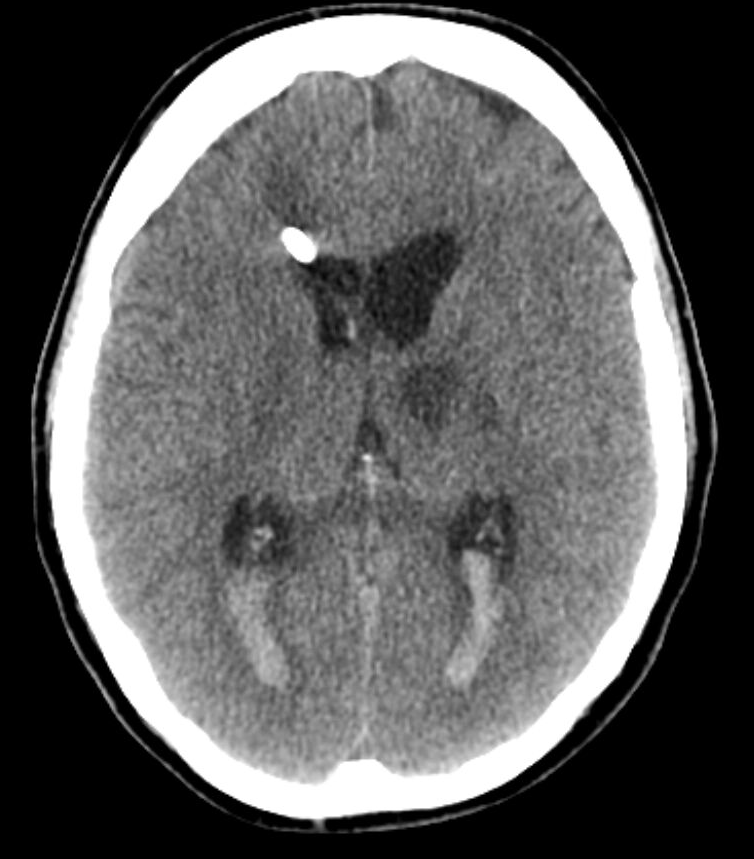 |
| --- | --- |

Major ischemic complication – Stent related 6, vertebral artery aneurysm

| 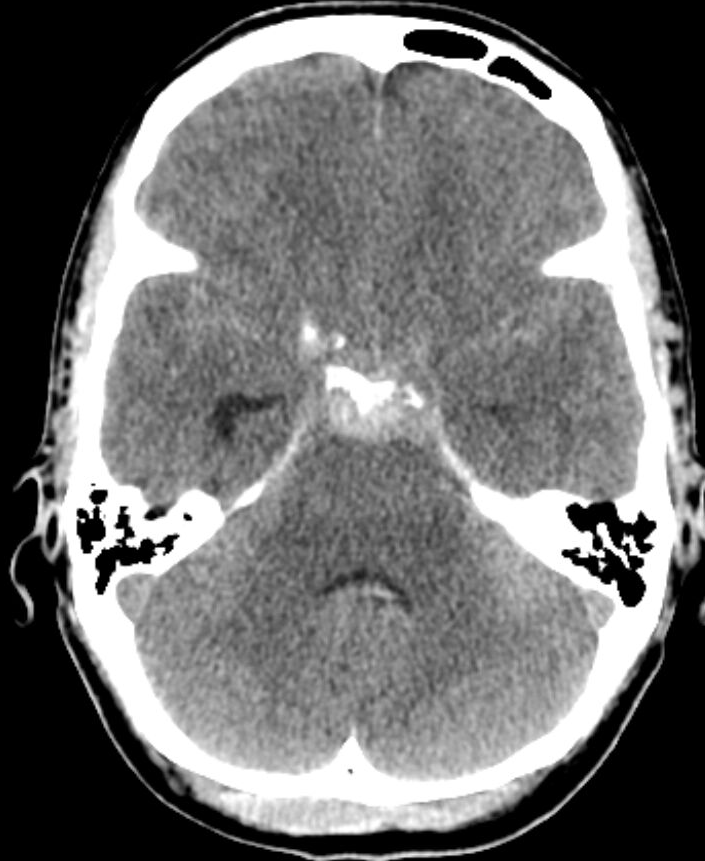 | 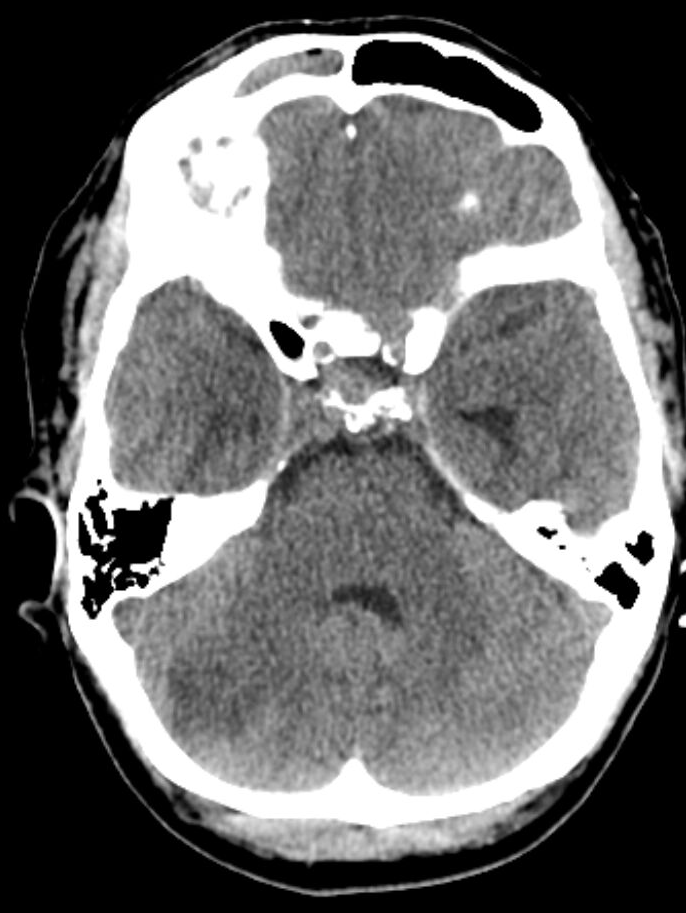 |
| --- | --- |

Major ischemic complication – Stent related 7, ICA aneurysm

| 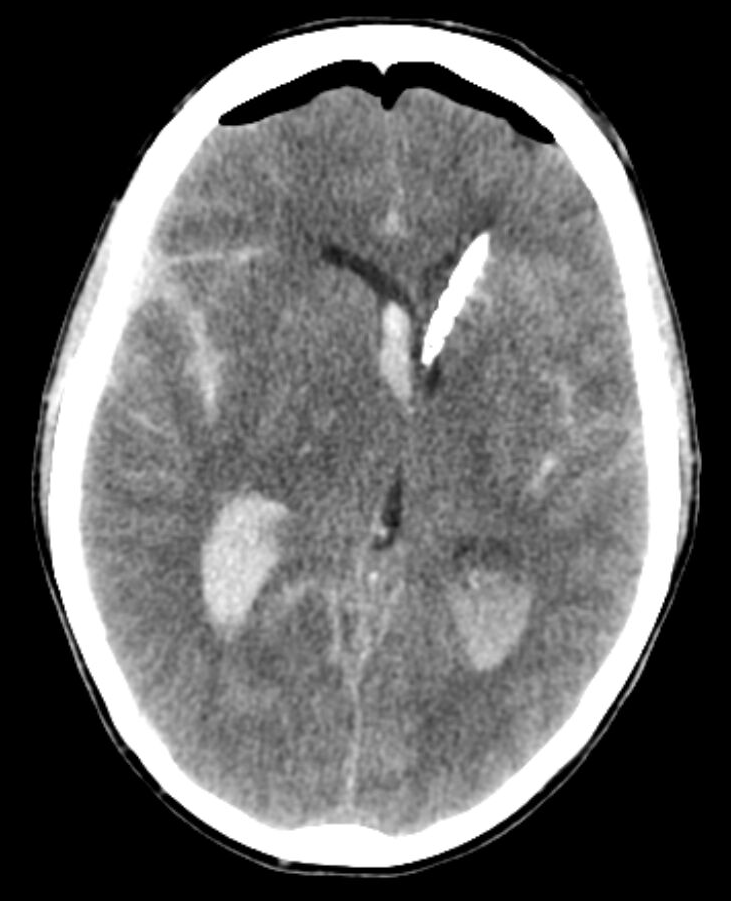 | 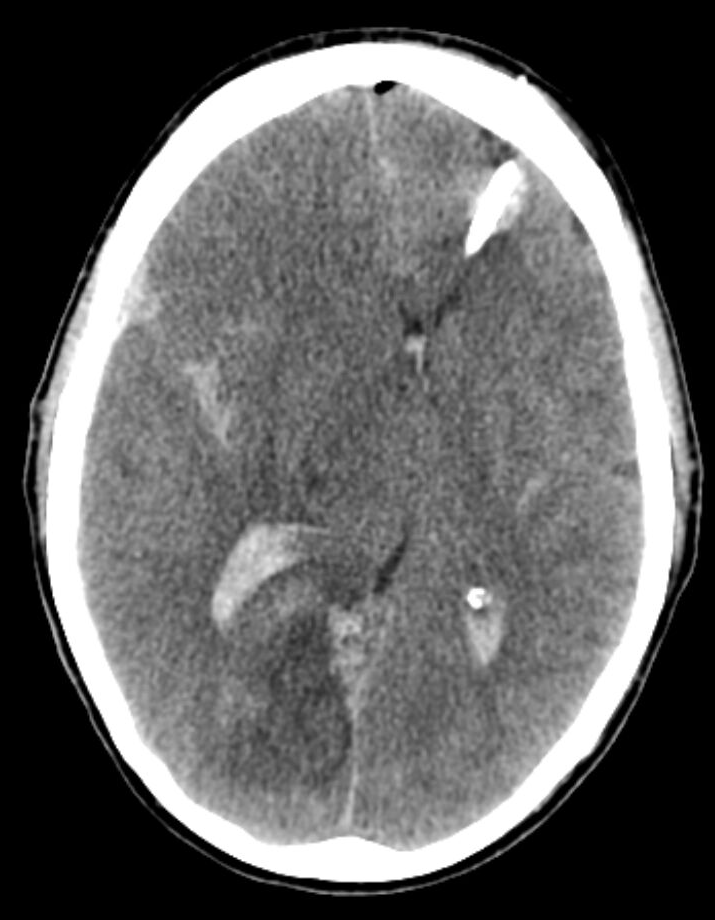 |
| --- | --- |

Major ischemic complication – Stent related 8, ICA aneurysm

| 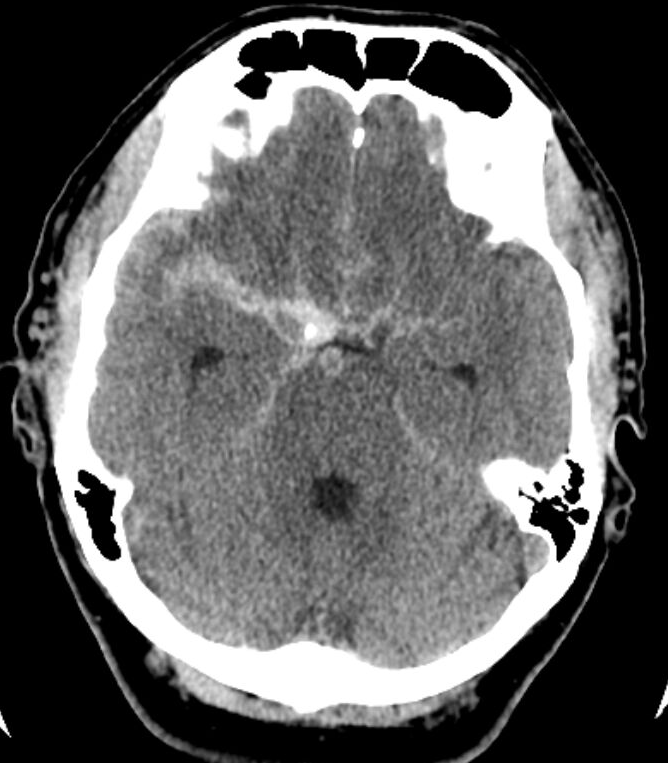 | 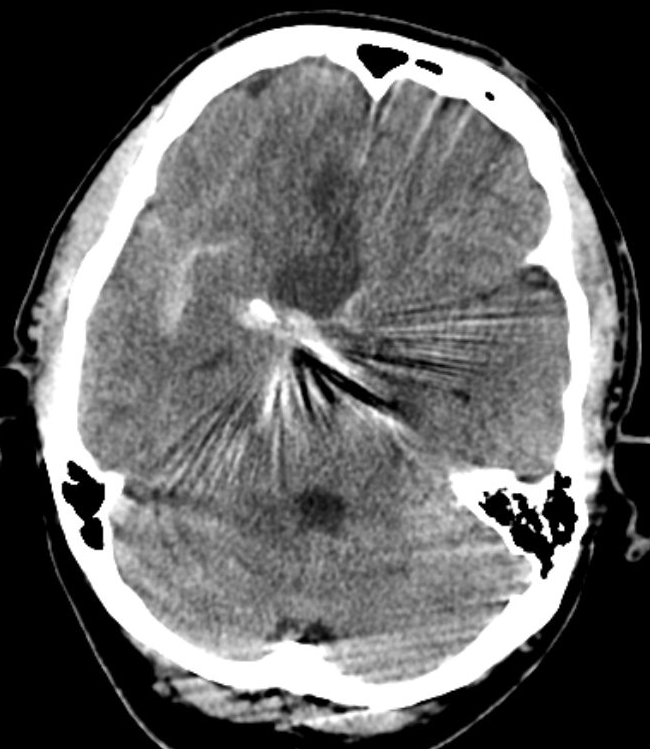 |
| --- | --- |

Major ischemic complication – Stent related 9, pericallosal aneurysm

| 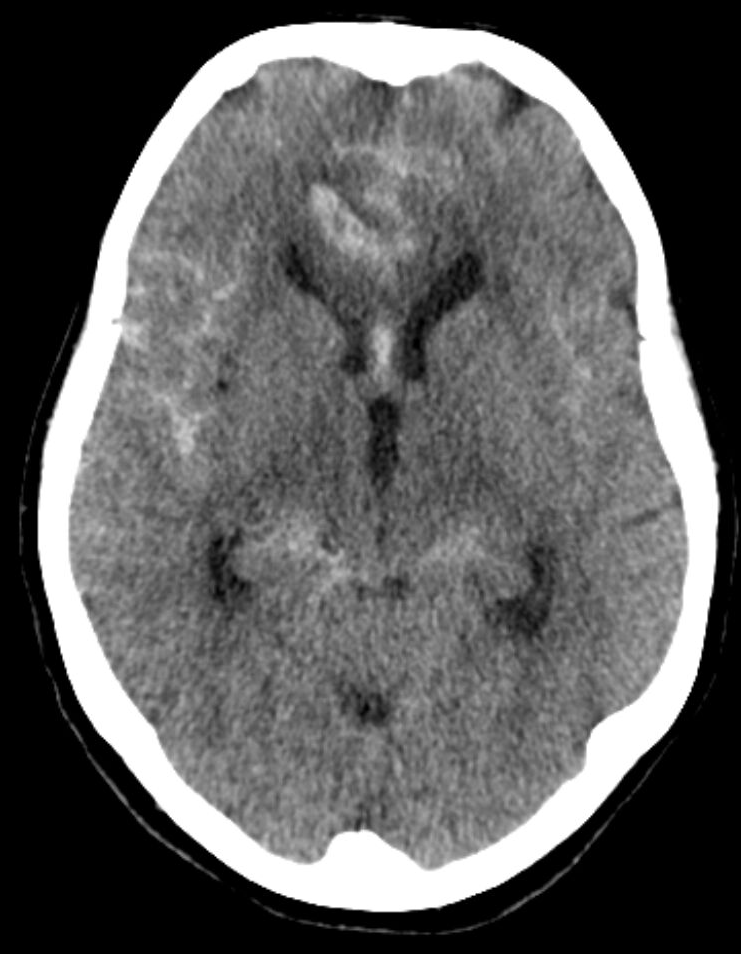 | 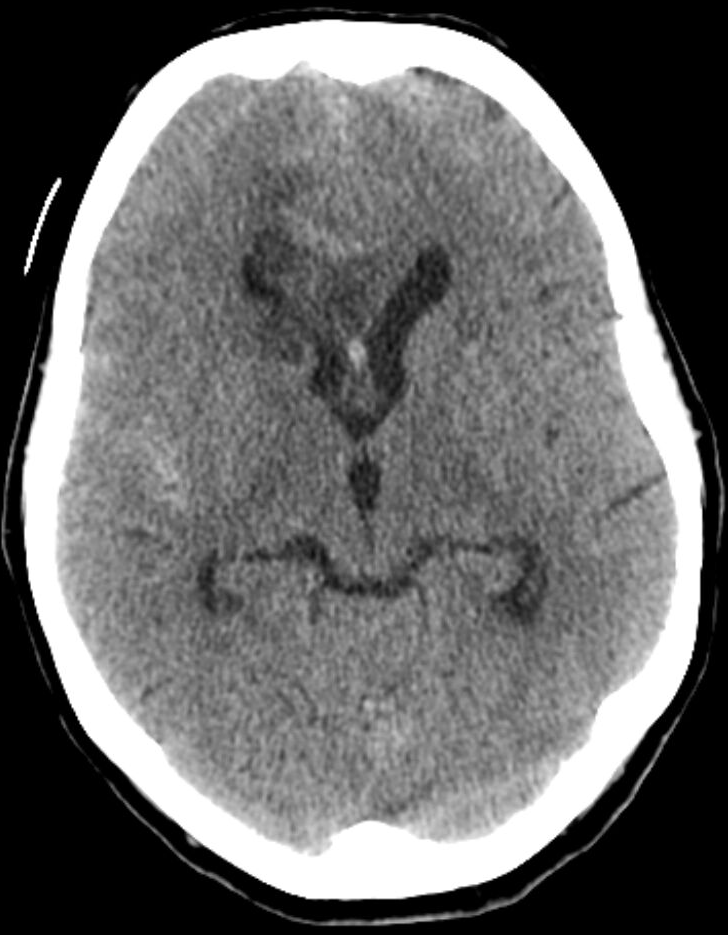 |
| --- | --- |

Major ischemic complication – Stent related 10, vertebral artery aneurysm

| 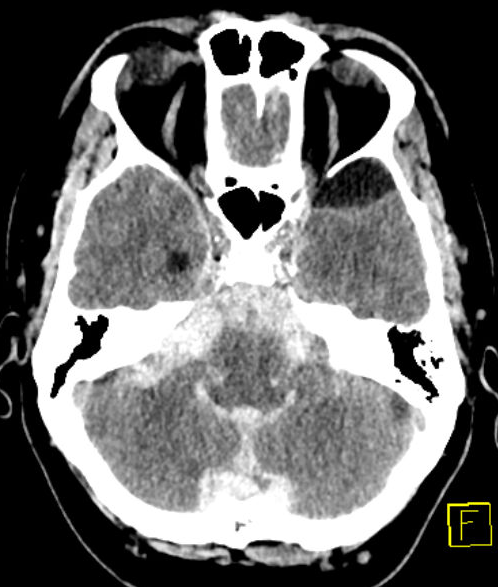 | 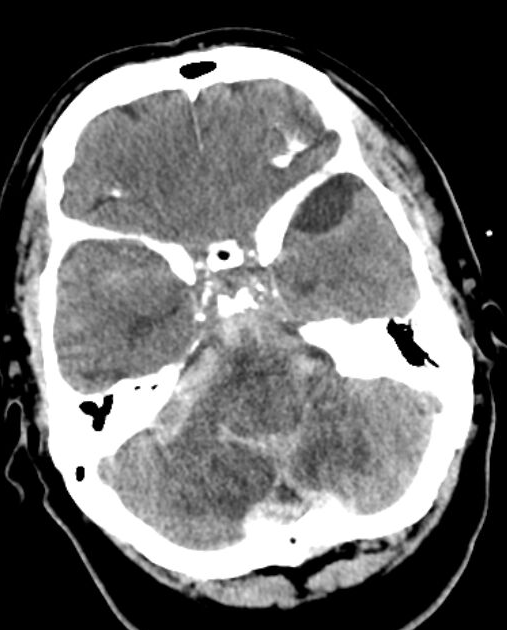 |
| --- | --- |

Minor ischemic complication – Stent related 1

| 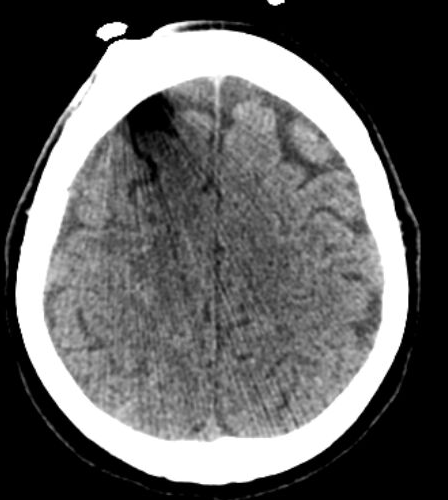 | 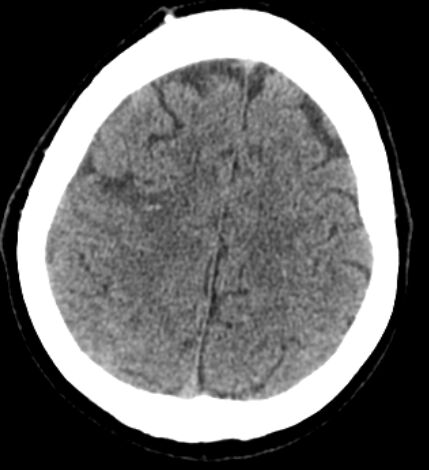 |
| --- | --- |

Minor ischemic complication – Stent related 2

| 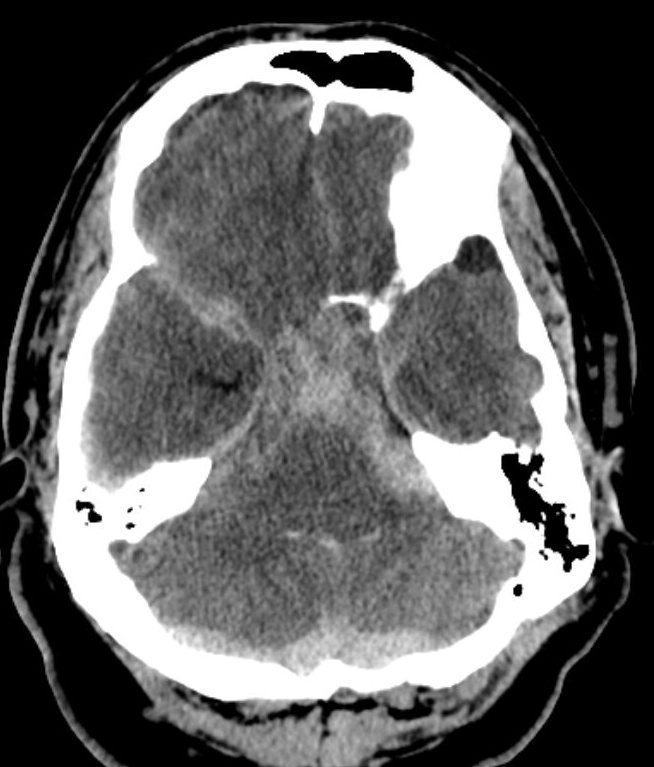 | 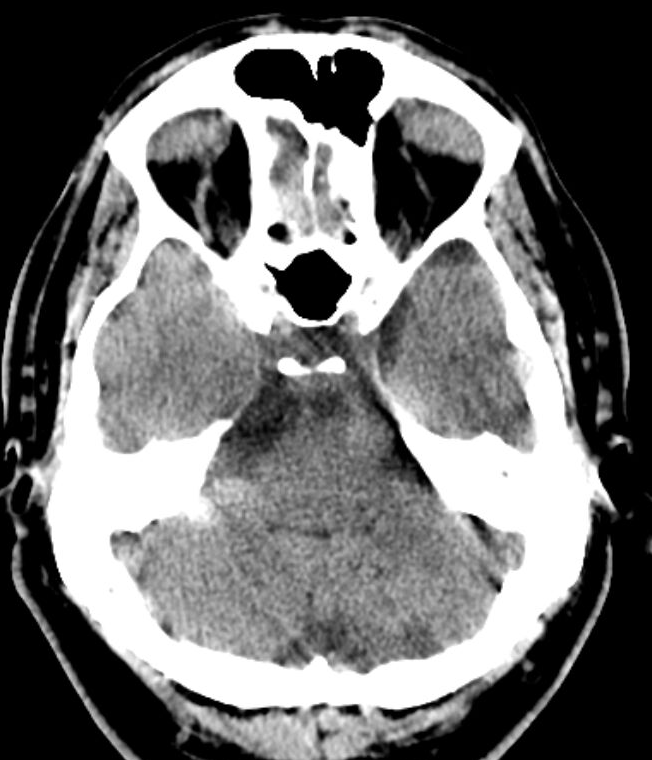 |
| --- | --- |

Minor ischemic complication – Stent related 3

| 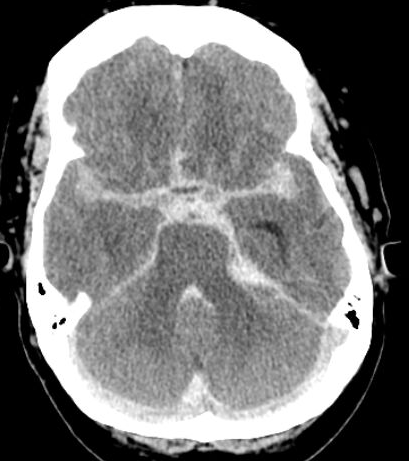 | 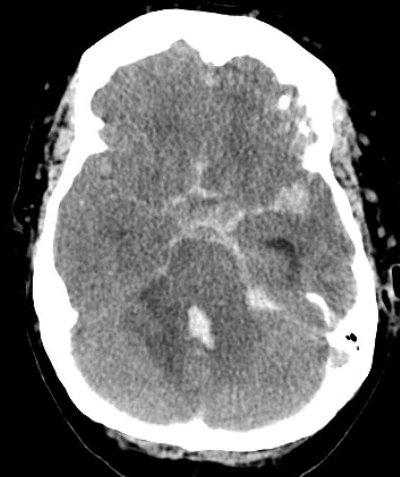 |
| --- | --- |
