# Supplementary material for: Flow diversion of ruptured intracranial aneurysms: a single-center study with a standardized antithrombotic treatment protocol
Source: Acta Neurochir (Wien). 2024 Mar 11;166(1):130. doi: 10.1007/s00701-024-06029-7 (PMC10927838; doi:10.1007/s00701-024-06029-7)
Supplement: Supplementary file 3 — Supplementary file3 (DOCX 23 KB) [file 701_2024_6029_MOESM3_ESM.docx]

| **Supplementary Table 1:** Used flow diverter stent models and manufacturers. | | |
| --- | --- | --- |
| **Stent brand** | **Pre-group** | **Post-group** |
| **Surpass®** (Stryker, Kalamazoo, Michigan, U.S.) | 10 | 7 |
| **Pipeline®** (Medtronic, Minneapolis, MN, U.S.) | 11 | 6 |
| **Silk®** (Balt Extrusion, Montmorency, France) | 1 | 0 |
| **Fred®** (Aliso Viejo, CA, U.S.) | 5 | 1 |
| Reported as total number of stents used. Stent brand does not indicate which version/generation of the stent was used. | | |

| **Supplementary Table 2:** Logistic regression model showing association between treatment after implementation of the protocol vs. before and treatment according to protocol vs. not. | | |
| --- | --- | --- |
| **Variable** | **OR (95% CI)** | **p-Value** |
| **Odds for unfavorable functional outcome at 6 months** | | |
| Post-group vs. pre-group | 0.90 (0.15–5.56) | 0.912 |
| Treated according-to-protocol vs. not | 0.38 (0.08–1.76) | 0.218 |
| **Odds for death within 6 months** | | |
| Post-group vs. pre-group | 0.69 (0.11–4.46) | 0.699 |
| Treated according-to-protocol vs. not | 0.57 (0.12–2.72) | 0.481 |
| Logistic regression models adjusted for age, sex, posterior circulation aneurysm, WFNS I–III vs. IV–V and modified Fisher I–II vs. III-IV. | | |
